# Monitoring global protein thiol-oxidation and protein *S-*mycothiolation in *Mycobacterium smegmatis* under hypochlorite stress

**DOI:** 10.1038/s41598-017-01179-4

**Published:** 2017-04-26

**Authors:** Melanie Hillion, Jörg Bernhardt, Tobias Busche, Martina Rossius, Sandra Maaß, Dörte Becher, Mamta Rawat, Markus Wirtz, Rüdiger Hell, Christian Rückert, Jörn Kalinowski, Haike Antelmann

**Affiliations:** 1 Institute for Biology-Microbiology, Freie Universität Berlin, D-14195 Berlin, Germany; 2 Institute for Microbiology, Ernst-Moritz-Arndt-University of Greifswald, D-17487 Greifswald, Germany; 3 Center for Biotechnology, Bielefeld University, D-33594 Bielefeld, Germany; 4 Department of Biology, California State University - Fresno, Fresno, CA 937401 United States; 5 Plant Molecular Biology, Centre for Organismal Studies Heidelberg, University of Heidelberg, Heidelberg, Germany; 6 Sinskey Lab, Department of Biology, Massachusetts Institute of Technology, Cambridge, MA USA

## Abstract

Mycothiol (MSH) is the major low molecular weight (LMW) thiol in Actinomycetes. Here, we used shotgun proteomics, OxICAT and RNA-seq transcriptomics to analyse protein *S*-mycothiolation, reversible thiol-oxidations and their impact on gene expression in *Mycobacterium smegmatis* under hypochlorite stress. In total, 58 *S*-mycothiolated proteins were identified under NaOCl stress that are involved in energy metabolism, fatty acid and mycolic acid biosynthesis, protein translation, redox regulation and detoxification. Protein *S*-mycothiolation was accompanied by MSH depletion in the thiol-metabolome. Quantification of the redox state of 1098 Cys residues using OxICAT revealed that 381 Cys residues (33.6%) showed >10% increased oxidations under NaOCl stress, which overlapped with 40 *S*-mycothiolated Cys-peptides. The absence of MSH resulted in a higher basal oxidation level of 338 Cys residues (41.1%). The RseA and RshA anti-sigma factors and the Zur and NrdR repressors were identified as NaOCl-sensitive proteins and their oxidation resulted in an up-regulation of the SigH, SigE, Zur and NrdR regulons in the RNA-seq transcriptome. In conclusion, we show here that NaOCl stress causes widespread thiol-oxidation including protein *S*-mycothiolation resulting in induction of antioxidant defense mechanisms in *M*. *smegmatis.* Our results further reveal that MSH is important to maintain the reduced state of protein thiols.

## Introduction

Eukaryotes and Gram-negative bacteria utilize glutathione (GSH) as their major low molecular weight (LMW) thiol. However, most Gram-positive bacteria do not produce GSH^1^. Instead, the Actinomycetes, including streptomycetes, mycobacteria and corynebacteria, synthesize mycothiol (AcCys-GlcN-Ins, MSH) as GSH-surrogate and major LMW thiol^1–3^. MSH functions in detoxification of ROS, alkylating agents, toxins, antibiotics, heavy metal stress and aromatic compounds^2, 4–7^. Apart from MSH, mycobacteria produce the histidine-derived alternative LMW thiol ergothioneine (EGT) and both, MSH and EGT are important to maintain redox and bioenergetics homeostasis and are required for virulence in *Mycobacterium tuberculosis* ^8^.

In previous studies, we have shown in *Corynebacterium glutamicum* that MSH is engaged in redox regulation and thiol-protection of proteins under hypochlorite stress by the formation of mixed disulfides, termed as *S*-mycothiolations^9^. The identified *S-*mycothiolated proteins function in many metabolic pathways, such as the central carbon metabolism, the biosynthesis of amino acids, nucleotides, thiamine and myo-inositol-1-phosphate as well in protein translation. Some *S-*mycothiolated proteins are conserved and abundant targets for *S*-thiolations across Gram-positive bacteria, including ribosomal proteins, GuaB, SerA and MetE^9, 10^. *S-*mycothiolated proteins include also antioxidant enzymes, such as peroxiredoxins (Tpx, Mpx) and methionine sulfoxide reductases (MsrA). The reduction of *S*-mycothiolated proteins is catalyzed by the mycoredoxin-1 (Mrx1)/MSH/mycothiol disulfide reductase (Mtr) pathway as well as by the thioredoxin (Trx)/thioredoxin reductase pathways that control the activity of Tpx, Mpx and MsrA *in vitro* ^11^. Mpx and MsrA form intramolecular disulfides and *S-*mycothiolations under H_2_O_2_ treatment *in vitro* and require both the Trx and Mrx1 pathways for regeneration^12, 13^. The Mrx1/Mtr/MSH pathway is also involved in reduction of the peroxiredoxin AhpE in *M*. *tuberculosis* ^14^.

Mycobacteria produce high levels of 20 mM MSH, but the impact of MSH to maintain the reduced state of protein thiols and its role in protein *S-*mycothiolation under oxidative stress are unknown. This knowledge about the role of MSH in redox modifications is particularly important since MSH plays an important role for virulence in *M*. *tuberculosis* ^15^. Thus, conserved S-mycothiolated proteins and major redox-switches in mycobacteria could be future drug targets to treat live-threatening tuberculosis disease. Here, we combined shotgun-proteomics, OxICAT and “*Voronoi redox treemaps*” to monitor protein *S-*mycothiolation and reversible thiol-oxidations and to analyze the role of MSH for the redox balance in the model bacterium *Mycobacterium smegmatis* under hypochlorite stress. Using RNA-seq transcriptomics, the regulatory impact of thiol-oxidation of NaOCl-sensitive transcription factors on the changes in gene expression was analyzed.

## Results

### *Mycobacterium smegmatis* tolerates high NaOCl concentrations resulting in strongly increased protein *S-*mycothiolation

**Figure 1:**
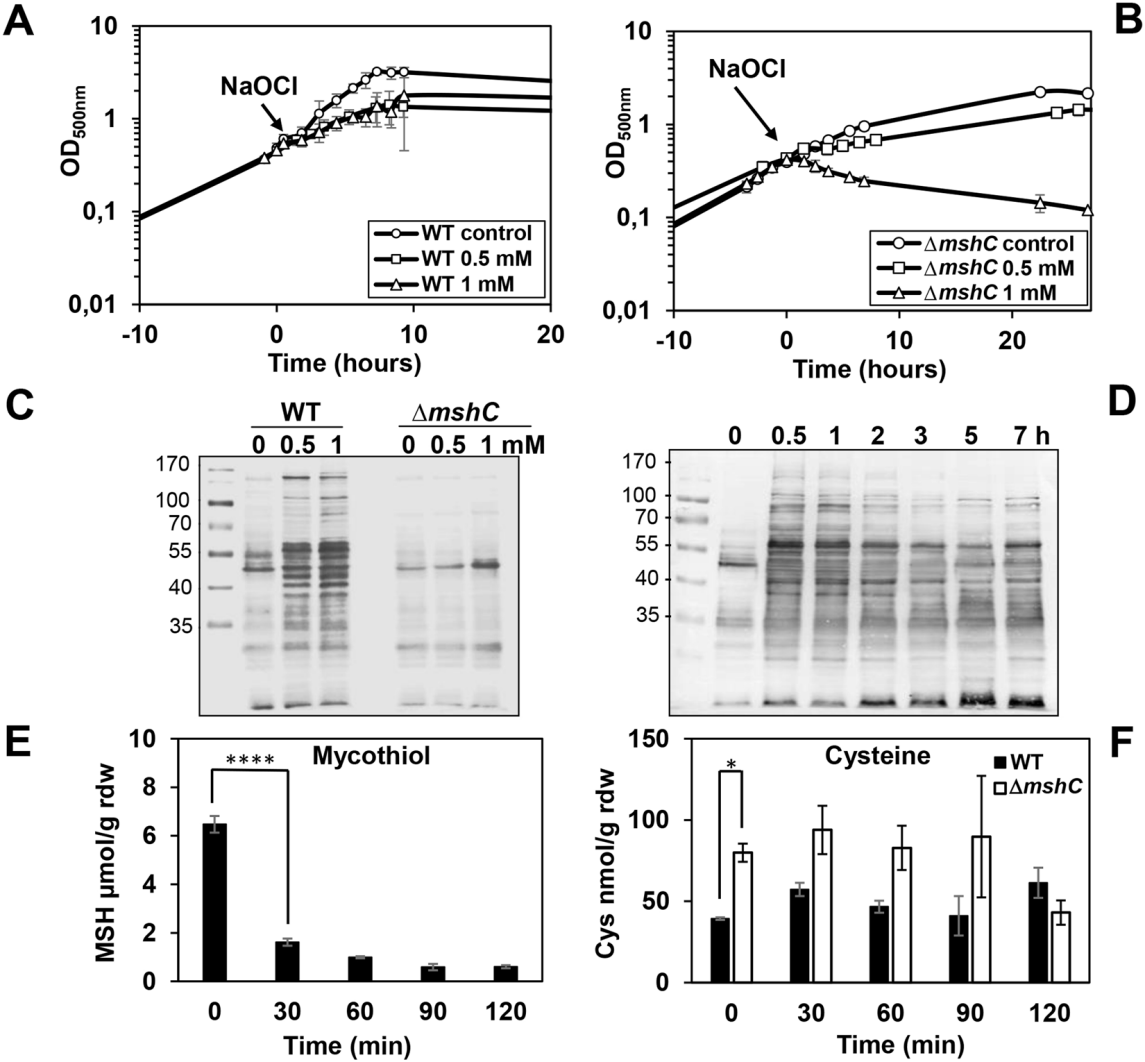
*M*. *smegmatis* tolerates high doses of 1 mM NaOCl leading to strongly increased protein *S*-mycothiolation and depletion of MSH in the thiol-metabolome. (**A**,**B**) The *M*. *smegmatis* wild-type and the Δ*mshC* mutant strains were cultivated in HdB minimal medium and exposed to sub-lethal concentrations of 0.5–1 mM NaOCl at an OD_500_ of 0.4. In contrast to the wild type, the ∆*mshC* mutant was unable to grow with 1 mM NaOCl. (**C**,**D**) Protein *S-*mycothiolation was increased in the wild type after exposure to 0.5–1 mM NaOCl stress as shown using non-reducing MSH-specific immunoblot analysis. (**E**) Thiol-metabolomics revealed the strong depletion of MSH in the wild type in response to 1 mM NaOCl stress indicating that MSH is used for protein *S-*mycothiolation. The MSH level decreased from 6.5 to 1.6 ± 0.25 µmol/g rdw after 30 min of NaOCl stress (One-way ANOVA, n = 15, *P* < 0.0001 for Co/NaOCl). (**F**) The Cys*-*levels in the control were calculated as 39.4 ± 1.33 nmol/g rdw in the wild type and 79.9 ± 9.75 nmol/g rdw in the ∆*mshC* mutant (Unpaired t-test, n = 6, p = 0.0173 for WT/∆*mshC* at t = 0 min). No significant changes in the Cys levels were measured after NaOCl stress in both strains (One-Way ANOVA, n = 15, *P* > 0.05 for WT and the *mshC* mutant Co/NaOCl).

We were interested to study the overall extent of protein *S*-mycothiolation under NaOCl stress in *M*. *smegmatis* since NaOCl was previously shown to cause strongly increased protein *S*-thiolations in several bacteria^1^. First, we determined the physiological NaOCl-concentration that reduced the growth-rate, but still was sub-lethal in *M*. *smegmatis* allowing recovery of growth. *M*. *smegmatis* wild type was grown in Hartman’s-de Bont minimal medium (HdB) with glycerol as carbon source and exposed to different concentrations of NaOCl stress during the exponential growth^16^. The sub-lethal NaOCl-concentration that reduced the growth rate was determined as 1 mM, while 500 µM did not affect the growth (Fig. 1A). Thus, *M*. *smegmatis* is able to survive and recover in growth after treatment with high doses of 1 mM NaOCl. To analyse the effect of MSH in this high NaOCl resistance, we compared the growth of the wild type with that of the *mshC* mutant. In contrast to the wild type, the *mshC* mutant was unable to grow with 1 mM NaOCl (Fig. 1B), which provides *bona fide* evidence for the role of MSH in the protection against NaOCl stress.

Using MSH-specific Western blot analyses, the extent of protein S-mycothiolation was analysed in a time-dependent manner after exposure to 1 mM NaOCl (Fig. 1C,D). NaOCl stress resulted in strongly increased levels of *S*-mycothiolated proteins in the *M*. *smegmatis* wild type already 30 min after NaOCl stress. The level of *S*-mycothiolated proteins was decreased again after seven hours of NaOCl exposure correlating with the resumed growth of cells. Thus, this strongly increased protein *S-*mycothiolation in *M*. *smegmatis* is consistent with the high MSH level as determined previously^17^.

Protein *S-*mycothiolation should result in a depletion of MSH in the metabolome and was analyzed using thiol-metabolomics (Fig. 1E). The intracellular MSH concentration was determined as 6 µmol/g raw dry weight (rdw) in untreated wild-type cells and decreased 5-fold after 30 min of NaOCl exposure. The MSH depletion in the thiol-metabolome confirms that MSH is used for protein *S-*mycothiolation under NaOCl stress. In contrast to MSH, the cysteine levels were low with 40–60 nmol/g rdw in the wild type and 80–90 nmol/g rdw in the *mshC* mutant (Fig. 1F). Thus, cysteine cannot compensate for the absence of MSH as LMW thiol in mycobacteria.

### Shotgun LC-MS/MS analysis identifies 58 *S-*mycothiolated proteins under NaOCl-stress conditions

**Figure 2:**
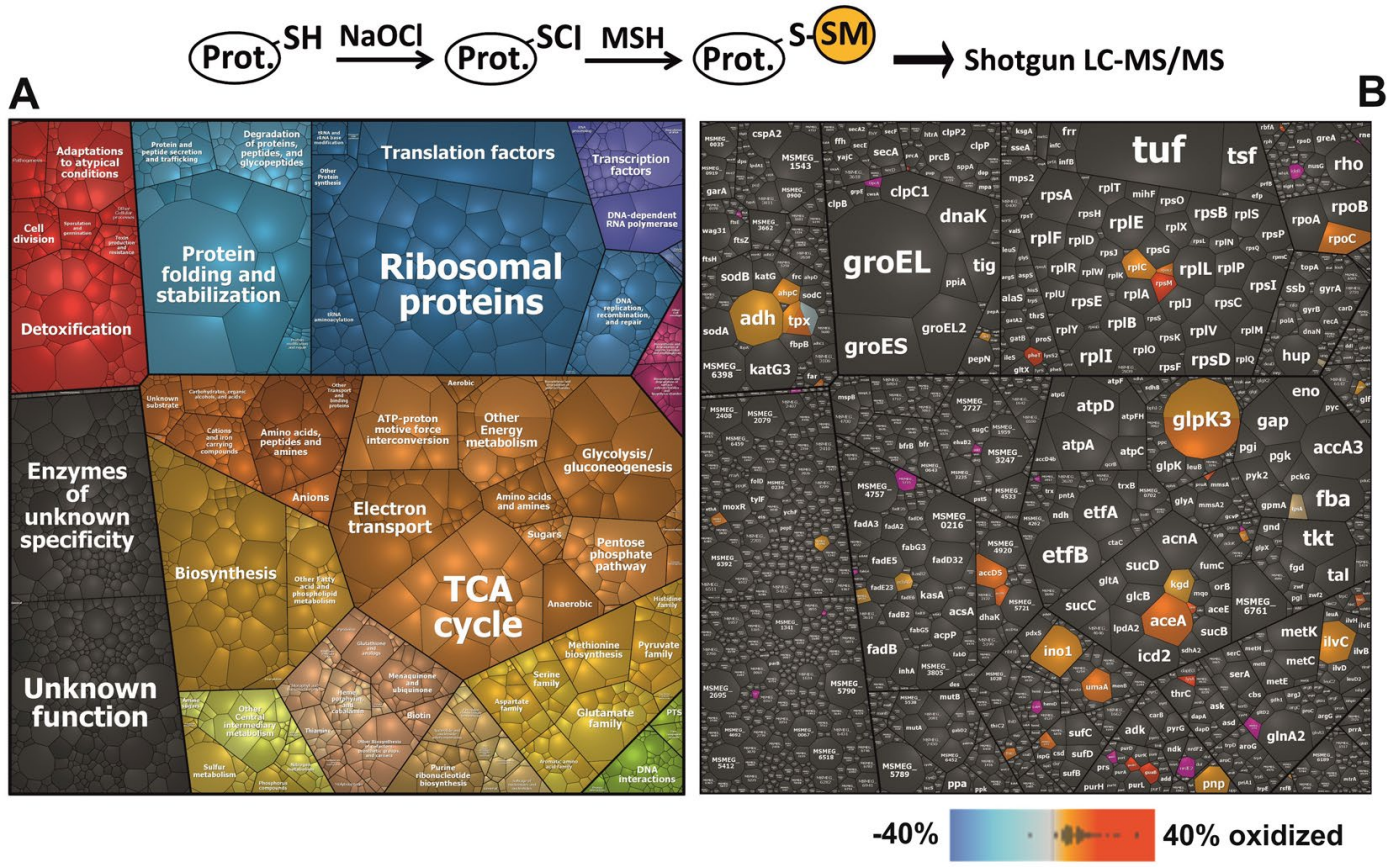
Voronoi treemaps show protein abundance of *S*-mycothiolated proteins identified in *M. smegmatis* under NaOCl stress using Orbitrap LC-MS/MS analysis. (**A**) The treemap legend shows the classification of the *M*. *smegmatis* proteome according to TIGRfam annotations. (**B**) The total spectral counts determine the cell size of each protein identified in the proteome dataset and classified according to TIGRfam. The identified 58 *S*-mycothiolated proteins are color-coded using an orange-red color gradient based on their Cys oxidation level as quantified by the OxICAT data (Tables 2, S3 and S4). Non-modified proteins are colored in grey and *S*-mycothiolated proteins that were not identified using the OxICAT approach are shown in pink.

Next, we used shotgun LTQ-Orbitrap LC-MS/MS analysis, to analyze *S*-mycothiolated Cys-peptides in the NEM-alkylated protein extracts of *M*. *smegmatis* under NaOCl stress based on their mass increase of +484 Da for MSH. In total, 58 proteins with *S-*mycothiolated Cys-peptides were identified (Table 1, Table S1A–C) which are visualized in *Voronoi treemaps* in relation to the total protein abundance of all proteins present in the proteome (Fig. 2). Among the identified *S-*mycothiolated proteins are known peroxiredoxins, such as Tpx, AhpC and OsmC that are *S-*mycothiolated at their active and/or resolving Cys residues (Table S1A,B). The inhibition of Tpx activity by *S-*mycothiolation and reactivation by the Mrx1/MSH/Mtr electron pathway was previously shown for Tpx of *C*. *glutamicum* ^9^. AhpC has been shown to function as peroxidase and peroxinitrite reductase together with AhpD, the dihydrolipoamide succinyl transferase (SucD) and the NADH-dependent dihydrolipoamide dehydrogenase (Lpd), thus linking the antioxidant response to regeneration of important enzymes of the intermediary metabolism^18^.

**Table 1:** Selected *S*-mycothiolated proteins of *Mycobacterium smegmatis* wild type and quantification of their % oxidation by OxICAT.

MSMEG-ID	Protein	Function	CysSSM peptide	% Ox NaOCl/co
**Antioxidant enzymes**				
MSMEG_4891	AhpC	AhpC peroxiredoxin	(K)DFTFVC_61_(+484)PTEIAAFGK(L)	6,70
MSMEG_2421	OsmC	OsmC family protein	(R)AVDQVC_116_(+484)TVGR(T)	10,49
MSMEG_3479	Tpx	Thiol peroxidase	(K)SVLLNIFPSVDTPVC_60_(+484)ATSVR(T)	11,57
			(K)AASSGATVLC_80_(+484)VSK(D)	−9,09
			(R)FC_93_(+484)GAEGIENVTTASAFR(S)	6,91
**Protein biosynthesis and quality control**				
MSMEG_1436	RplC	50 S ribosomal protein L3	(R)RPGSIGGC_154_(+484)ATPGR(V)	7,32
MSMEG_1521	RpsM	30 S ribosomal protein S13	(R)KIEIGC_86_(+484)YQGLR(H)	21,77
MSMEG_6895	RpsR2	30 S ribosomal protein S18	(R)VTGNC_57_(+484)VQHQR(D)	10,66
MSMEG_0839	Lon1	ATP-dependent protease Lon	(R)IIDC_72_(+484)QNLGANR(Y)	25,70
MSMEG_0832	Def	Peptide deformylase	(R)LFVYDC_68_(+484)APTR(G)	5,76
**Transcriptional regulation**				
MSMEG_2750	IdeR	Iron-dependent repressor IdeR	(R)LLVDVIGLPWEDVHAEAC_102_(+484)R(W)	—
MSMEG_4953	TetR2	TetR-family transcriptional regulator	(R)LIDAAETC_21_(+484)LR(A)	—
MSMEG_0227	TetR1	TetR-family transcriptional regulator	(R)LTAILLGPEPGTAC_143_(+484)R(V)	—
**Biosynthesis of cofactors**				
MSMEG_0913	UmaA	Methoxy mycolic acid synthase 1	(K)LDLKPGMTLLDVGC_76_(484)GWGGALER(A)	10,46
MSMEG_6904	Ino1	Inositol-3-phosphate synthase	(R)VAIVGVGNC_18_(+484)ASSLVQGVQYYR(N)	6,31
MSMEG_0793	ThiG	Thiazole synthase	(R)LGIAALPNTAGC_75_(+484)R(G)	10,25
**Energy metabolism**				
MSMEG_6242	Adh2	Putative glycerol dehydrogenase	(R)AISEHIQDDWC_398_(+484)TPGNPR(E)	4,94
MSMEG_6759	GlpK3	Glycerol kinase	(K)NGLLTTVC_294_(+484)YR(L)	10,73
			(R)ATLESIC_389_(+484)YQSR(D)	5,67
MSMEG_3086	TpiA	Triosephosphate isomerase	(R)VAGAADAQEVC_192_(+484)K(A)	2,04
MSMEG_0911	AceA	Isocitrate lyase	(K)NGLEPC_268_(+484)IAR(A)	11,36
MSMEG_5676	CitA	Citrate synthase	(R)TIDEC_143_(+484)PTVTAR(F)	14,23
MSMEG_5049	Kgd	2-oxoglutarate metabolism enzyme	(R)SSEYC_695_(+484)TDVAK(M)	4,60
**Metabolism of fatty acids and phospholipids**				
MSMEG_5639	EchA6	Enoyl-CoA hydratase	(R)NALNC_26_(+484)ELVDSLR(E)	4,73
MSMEG_0531	MSMEG_0531	Acyl-CoA dehydrogenase	(R)AAYEYALDYAC_285_(+484)QR(E)	4,50
MSMEG_6208	MSMEG_6208	Acyl-CoA thioesterase	(R)DGDVFC_21_(+484)IREPEPNTIER(L)	—﻿
MSMEG_1813	AccD5	Propionyl-CoA carboxylase beta chain	(R)VEGRPVGIVANQPTQFAGC_356_(+484)LDINASEK(A)	11,49
MSMEG_4329	AccD6	Acetyl/propionyl-CoA carboxylase	(R)LGGC_294_(+484)LNSESAEK(S)	12,96
**Metabolism of nucleotides**				
MSMEG_2299	NrdE2	Ribonucleoside-diphosphate reductase	(K)ITHSNLC_380_(+484)SEILQVSTPSEFNDDLSYAK(V)	—
MSMEG_1602	GuaB	Inosine-5′-monophosphate dehydrogenase	(K)VGVGPGSIC_325_(+484)TTR(V)	19,34
MSMEG_3634	GuaB2	Inosine-5′-monophosphate dehydrogenase	(K)VGVGPGAmC_302_(+484)TTR(M)	33,37
MSMEG_2656	Pnp	Polyribonucleotide nucleotidyltransferase	(K)ALC_248_(+484)AAQQELADR(A)	5,67

The *M*. *smegmatis* wild type was exposed to 1 mM NaOCl for 30 min and 58 *S*-mycothiolated proteins were identified using shotgun LC-MS/MS analysis using the Scaffold proteome software based on the mass increase of 484 Da (+MSH) at Cys peptides. The table lists for 29 selected mycothiolated proteins the MSMEG-ID, the protein name, function and the S-thiolated Cys peptide sequence. The OxICAT data were extracted from Tables S3 and S4 for the *S*-mycothiolated Cys peptides. The full table of the 58 *S*-mycothiolated proteins is presented in Table S1A,B.

Interesting *S*-mycothiolated proteins include further transcriptional regulators that could be redox-controlled by protein *S*-mycothiolation, such as global regulator for iron uptake of the DtxR-family (IdeR) that was *S-*mycothiolated at Cys102, its primary iron-binding site (Fig. S1A). In *Corynebacterium diphtheriae*, the Cys-Asp mutant in the related DtxR-repressor was incompatible in DNA-binding to its target operators^19^. Apart from DtxR, two unknown TetR-family regulators (MSMEG_4953 and MSMEG_0227) were identified as *S-*mycothiolated transcription factors. Future studies should elucidate whether these transcriptional regulators function as redox-switches under NaOCl stress to control their specific target genes.

**Figure 3:**
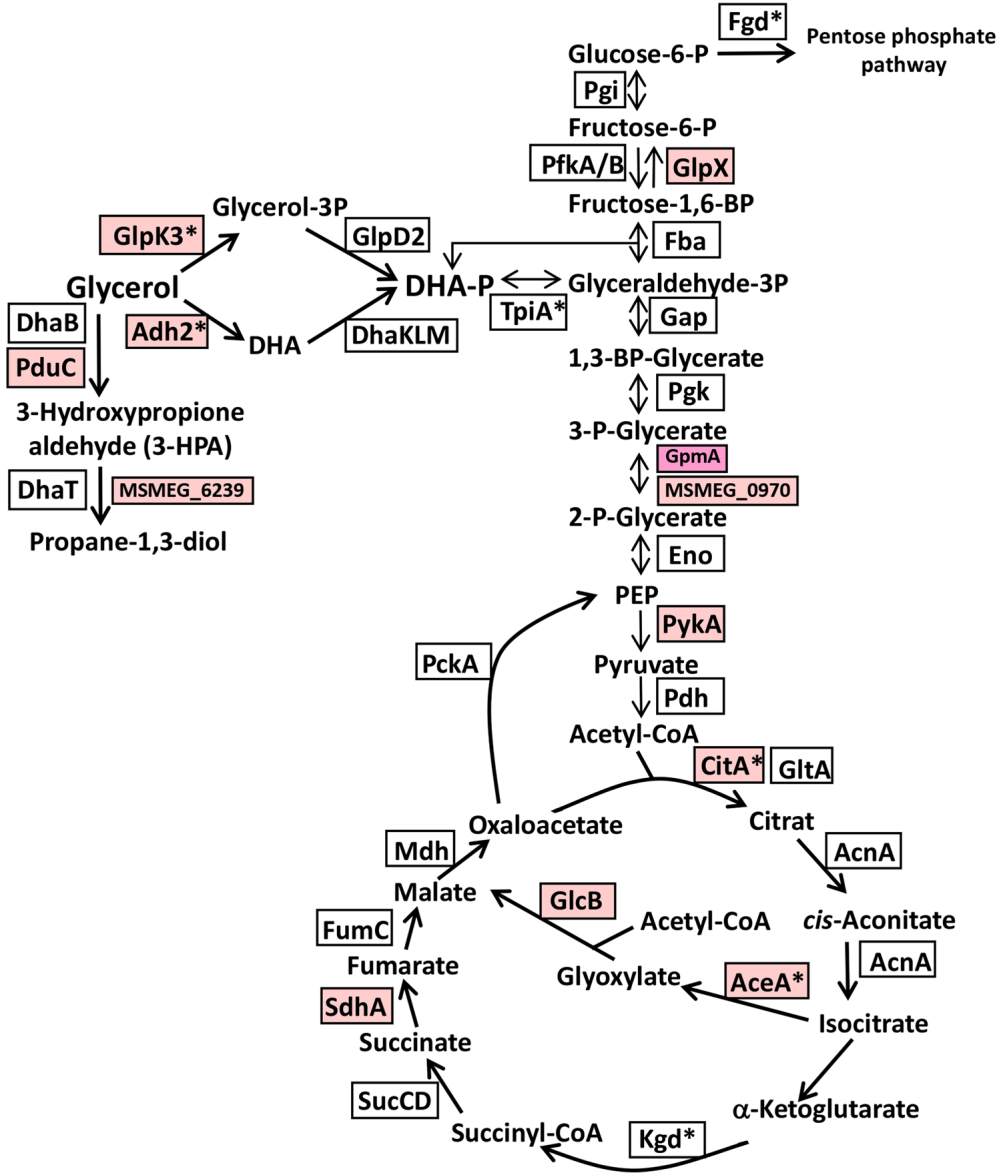
Schematics of the glycerol catabolism, glycolysis, TCA cycle, glyoxylate cycle and gluconeogenesis in *M. smegmatis* highlighting NaOCl-sensitive thiol-switches. The reversibly oxidized NaOCl-sensitive enzymes are color-coded in light and dark pink indicating 10% and 20% thiol-oxidation increase under NaOCl stress, respectively. The *S*-mycothiolated proteins are labelled with an asterisk. The pathways of the glycerol catabolism include the aerobic oxidation of glycerol to dihydroxyacetone-phosphate (DHA-P) and the propane-diol-pathway that are catalyzed by (1) the glycerol kinase (GlpK3 or MSMEG_6759) and glyceraldehyde dehydrogenase (GlpD2), (2) the glycerol dehydrogenase (Adh2 or MSMEG_6242) and dihydroxyacetone kinase complex (DhaKLM) and (3) the B12-dependent glycerol dehydratase (DhaB or MSMEG_1546-49) and propane-1,3-diol-dehydrogenase (MSMEG_6239). DHAP enters the glycolysis, TCA and glyoxylate shunt and gluconeogenesis for energy and biomass production. The gluconeogenesis enzymes include GlpX (fructose-1,6-Bis-phosphatase) and PckA (PEP-carboxykinase), while Pgi (glucose-6-phosphate isomerase), Fba (fructose-bisphosphate aldolase), Gap (glyceraldehyde-3-phosphate dehydrogenase), Pgk (phosphoglycerate kinase), GpmA and MSMEG_0970 (phosphoglycerate mutase) and Eno (enolase) are involved in both glycolysis and gluconeogenesis. The glyoxylate shunt includes the isocitrate lyase (AceA) and malate synthase (GlcB).

The largest group of *S*-mycothiolated proteins functions in energy metabolism that include abundant enzymes of the different routes of glycerol catabolism, glycolysis, the glyoxylate shunt and gluconeogenesis (MSMEG_6759 or GlpK3, MSMEG_6242 or Adh2, TpiA, CitA, AceA, Kgd) (Fig. 3, Tables 1, S1 and S2). Since *M*. *smegmatis* was grown with glycerol as sole carbon and energy source, the glycerol kinase GlpK3 and the glycerol dehydrogenase MSMEG_6242 (Adh2) are abundant *S-*mycothiolated proteins and specify two branches of the glycerol catabolic pathways (Fig. 3)^20^. GlpK3 uses ATP for phosphorylation of glycerol to glycerol-3-phosphate which is converted to dihydroxyacetone phosphate (DHAP) by the glycerol-3-phosphate dehydrogenase MSMEG_6761 (GlpD2) (Table S1D). The Adh2 enzyme (MSMEG_6242) reduces glycerol to DHA which is then phosphorylated by the DHA kinase (DhaKLM) to DHAP. *S-*mycothiolation of GlpK3 and Adh2 could prevent glycerol degradation under NaOCl stress to save the source of carbon and energy.

We further identified the isocitrate lyase AceA and the myo-inositol-1-phosphate synthase Ino1 as abundant *S-*mycothiolated proteins in *M*. *smegmatis*. AceA is the key enzyme of the glyoxylate bypass of the TCA cycle (Fig. 3) and required for growth on fatty acids in *M*. *tuberculosis* to enable the use of carbon for biomass production via gluconeogenesis^21^. AceA inhibition and protection by *S-*mycothiolation under oxidative stress could be important for survival of mycobacteria. Ino1 functions in MSH and phosphatidylinositol biosynthesis and was *S-*mycothiolated also in *C*. *glutamicum* (Fig. S1B).

Furthermore, enzymes involved in the biosynthesis of fatty acids as precursors for mycolic acids are essential in *M*. *tuberculosis* and abundant targets for *S*-mycothiolation in *M*. *smegmatis* (Fig. 2)^22, 23^. Among the *S*-mycothiolated proteins are acetyl-CoA carboxylases involved in the carboxylation of acetyl-CoA to malonyl-CoA as first step of the fatty acid biosynthesis (AccD5 and AccD6), the enoyl-CoA hydratase (EchA6), the methoxy mycolic acid synthase (UmaA), the acyl-CoA dehydrogenase (MSMEG_0531) and the acyl-CoA-thioesterase (MSMEG_6208). UmaA is *S*-mycothiolated at the conserved Cys76 that is required for *S*-adenosylmethionine binding.

*S*-mycothiolated enzymes participate in nucleotide biosynthesis, such as PurC, Pnp, NrdE2 and the conserved inosine-5′-monophosphate (IMP) dehydrogenases GuaB and GuaB2. Both GuaB homologs are *S*-mycothiolated at their conserved active sites, Cys325 and Cys302, respectively, that form the thioimidate intermediate (Fig. S1C). Other targets for *S-*mycothiolation are biosynthesis enzymes for thiamine (ThiG, MSMEG_4827), cobalamin (CobN), iron sulfur-cluster assembly (YfhF2) and translation proteins, such as ribosomal proteins (RplC, RpsM, RpsR2) and amino acyl tRNA synthetases (GatC, PheT). RplC and RpsM are *S*-mycothiolated at their conserved Cys154 and Cys86, respectively. The conservation of ribosomal proteins as targets for *S*-thiolation suggests an inhibition of protein biosynthesis under oxidative stress.

In previous studies, we have identified *S*-cysteinylated proteins in the absence of bacillithiol and mycothiol^9, 10^. Here, we detected only 11 *S*-cysteinylated proteins in the *mshC* mutant (Table S1A), including Tpx, AccD5, AccD6, CitA, RplC, RpsM and RpsR2 that were *S*-mycothiolated in the wild type. Thus, *S*-cysteinlyation cannot compensate for the loss of MSH in protein protection and redox-regulation. Mycobacteria also produce ergothioneine (EGT) as histidine-derived LMW thiol that is important to maintain redox and bioenergetics homeostasis in *M*. *tuberculosis* ^8, 17, 24^. However, we did not found EGT-mixed disulfides in the *mshC* mutant since these are probably instable modifications and escape the identification using mass spectrometry.

### Quantification of the redox status of 1098 Cys residues by OxICAT and visualization of the thiol-oxidation levels using “*Voronoi redox treemaps”*

The shotgun proteomics method identified only 58 S-mycothiolated proteins. However, this method is not quantitative due to the unstable nature of the MSH modification. Thus, we used the quantitative thiol-redox proteomics approach OxICAT to determine more comprehensively the thiol-oxidation state of all Cys residues including those that are S-mycothiolated in the *M*. *smegmatis* wild type. In addition, the *mshC* mutant was included in the OxICAT analysis^25, 26^ to reveal the role of MSH for the thiol- redox state of proteins in *M*. *smegmatis*.

The principle of the OxICAT method relies on the differential labelling of reduced and oxidized thiol-residues using isotope-coded affinity tags (ICAT)^25, 26^. Reduced Cys residues are labelled with light ^12^C-ICAT, followed by reduction of reversible thiol-oxidations (e.g. protein disulfides and *S*-thiolations) and subsequent labelling of previously oxidized thiols with heavy ^13^C-ICAT reagent. Light and heavy ICAT-labeled peptide pairs show a mass difference of 9 Da after separation using mass spectrometry. The percentage of thiol-oxidation for each Cys-peptide is calculated based on the intensity of the heavy ICAT-labeled Cys-peptide in relation to the total intensity of the light and heavy-ICAT-labelled Cys-peptides^25, 26^.

**Figure 4:**
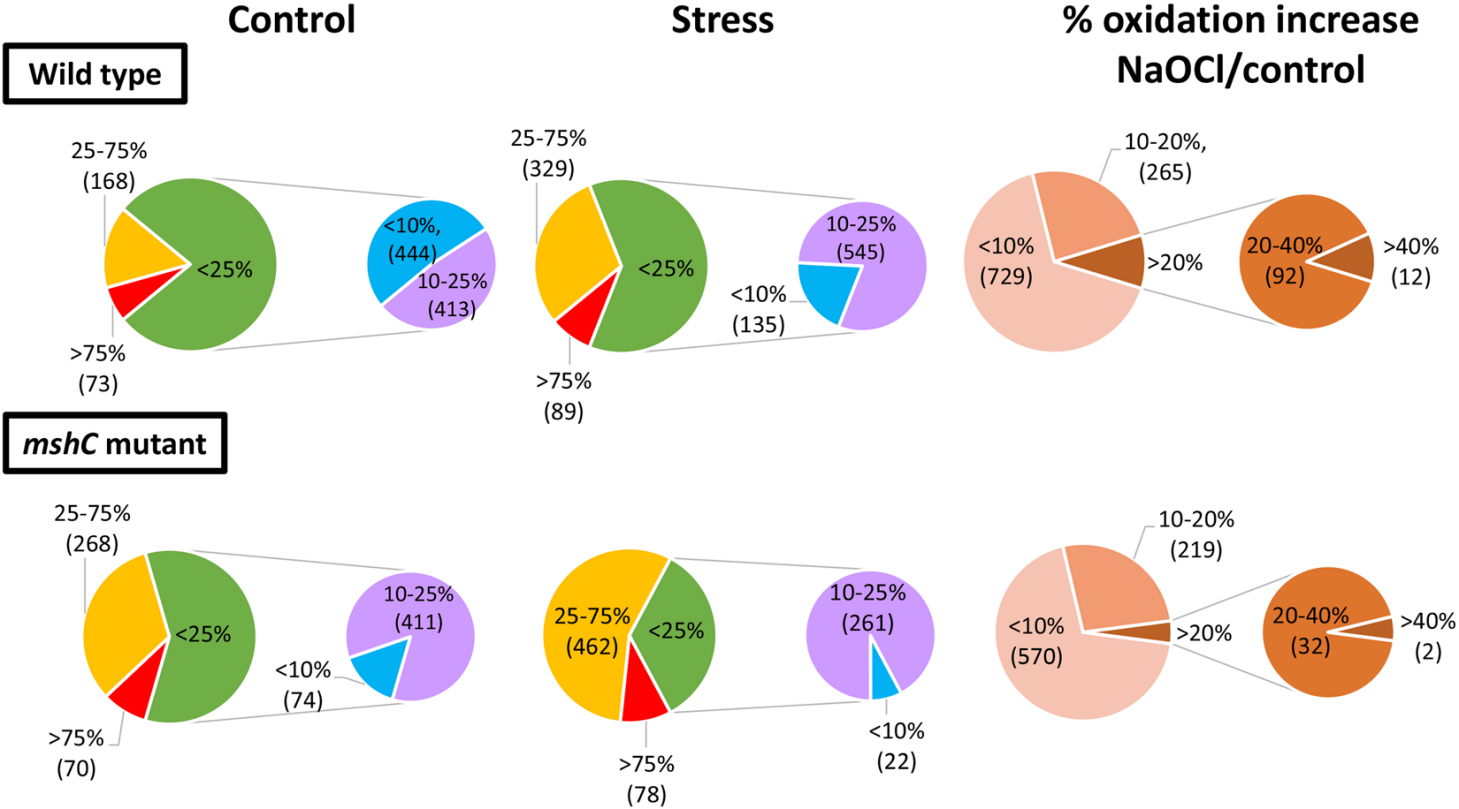
Overview of the percentages of thiol-oxidation levels of all Cys peptides identified in the redox proteome of the *M. smegmatis* wild type and the *mshC* mutant under control and NaOCl stress as revealed by OxICAT. Reduced Cys peptides with a <25% oxidation are shown in green including those <10% oxidized (blue) and 10–25% oxidized (magenta). Cys peptides with an oxidation degree of >25% and >75% are shown in yellow and red, respectively. The percentage of thiol-oxidation increase is shown with an orange-brown color gradient including Cys peptides with 10–20% and >20% increased oxidation by NaOCl stress. The *mshC* mutant shows a higher basal level oxidation in the control that resembles that of the wild type after NaOCl stress.

**Figure 5:**
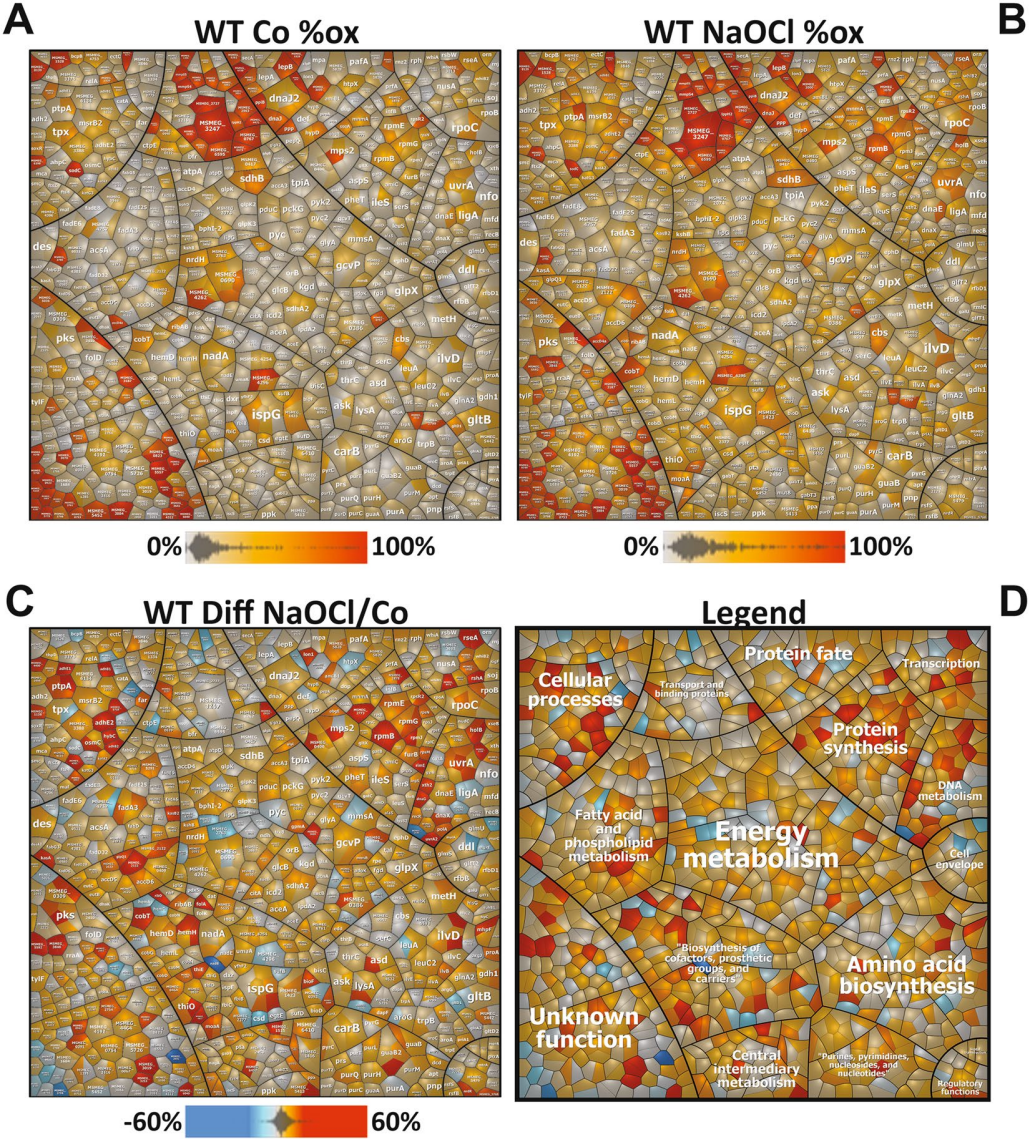
Voronoi redox treemaps show the percentages of thiol-oxidation levels of all Cys-peptides identified in the redox proteome of the *M. smegmatis* wild type. The “*Voronoi redox treemaps*” show the percentages of thiol-oxidations of 1098 Cys-residues identified using OxICAT in the wild type control (**A**) and 30 min after exposure to 1 mM NaOCl stress (**B**). The grey-yellow-red color gradient denotes 0–100% oxidation. The *Voronoi redox treemap* in (**C**) visualizes the percentages of oxidation changes under NaOCl stress using a blue-red color gradient ranging from −60 to +60% oxidation. The treemap in (**D**) is used as legend for the functional classification of the proteins displayed in (**C**). The treemaps are generated based on the OxICAT data presented in Table S4 using the Paver software (Decodon) and proteins were classified according to the *M*. *smegmatis* TIGRfam annotation.

Using OxICAT, the percentages of thiol-oxidation levels were quantified for 1098 Cys residues under control and NaOCl stress in *M*. *smegmatis* wild-type cells (Tables 2, S2–S4; Fig. 4). The Cys-peptides were color-coded according to their percentages of thiol-oxidations and visualized in “*Voronoi redox treemaps*” that are based on the TIGRfam classification (Fig. 5A–D). In the wild-type control, 857 Cys residues (78.1%) showed an oxidation state of <25%, that included 444 Cys residues (40.4%) with <10% oxidation. This indicates that the majority of all detected thiols are reduced under non-stress conditions in *M*. *smegmatis* (Fig. 5A). A minor part of 241 Cys residues (21.8%) had higher oxidation levels of >25% in the control. These basal level oxidized proteins belong to membrane proteins and ABC transporter components involved in transport functions, protein secretion, folding and quality control.

**Table 2:** Selected NaOCl-sensitive proteins with >10% increased thiol-oxidations under NaOCl stress in *M*. *smegmatis* as revealed using the OxICAT method.

Locus tag	Gene name	Protein function	Cys (*a*, *b*)	Buried/Exposed (*d*)	OxICAT Wild type			OxICAT Δ*mshC*		
					% Diff NaOCl/Co (*e*)	% ox Co (*f*)	% ox NaOCl (*f*)	% Diff NaOCl/Co (*e*)	% ox Co (f)	% ox NaOCl (*f*)
**Detoxification and adaptation to atypical environments**										
MSMEG_0127	adhE1	Alcohol DH, zinc-containing	48*	B	32,0	13,5	45,5	13,8	19,5	33,3
MSMEG_0217	adhB	Alcohol DH, zinc-containing	105*	B	39,1	20,1	64,8			
MSMEG_5866	adhB2	Alcohol DH, zinc-containing	106*	B	35,7	20,9	55,2	16,9	28,8	45,7
MSMEG_4340	adhE2	Alcohol DH, zinc-containing	107*	B	33,1	23,8	54,2	15,3	34,8	50,0
			145	B	34,6	18,0	43,8	18,5	28,7	47,2
MSMEG_1138	MSMEG_1138	Alcohol DH, zinc-containing	113*	B	26,0	16,5	43,8			
MSMEG_4400	MSMEG_4400	Alcohol DH, zinc-containing	65	B	27,8	6,4	34,1			
MSMEG_1977	MSMEG_1977	Alcohol DH, zinc-containing	39*	B	22,8	14,5	30,0	11,9	12,6	27,5
MSMEG_0595	MSMEG_0595	Fe-S oxidoreductase	142*	B	13,9	12,4	23,4	7,6	30,2	37,8
MSMEG_0690	MSMEG_0690	Fe-S oxidoreductase	637*	B	11,5	22,7	38,8	8,2	38,5	45,2
MSMEG_0768	MSMEG_0768	Rhodanese domain protein	83*	B	42,6	17,2	55,5	23,0	30,5	53,8
MSMEG_6425	MSMEG_6425	Rhodanese-domain protein	66*	B	14,0	7,1	20,9	7,8	13,1	22,7
MSMEG_1416	MSMEG_1416	Pyridine nucleotide-disulfide oxidoreductase	159	B	11,4	12,0	20,3	12,5	14,3	26,7
MSMEG_1566	MSMEG_1566	Oxidoreductase	122	B	14,2	15,1	24,9			
MSMEG_2263	hybC	Cytochrome-c3 hydrogenase	58	B	29,3	21,7	49,7	3,8	30,7	37,9
MSMEG_2297	nrdH	Glutaredoxin	11*	B	14,2	45,7	56,1	15,0	57,7	69,2
MSMEG_2421	osmC	OsmC family protein	48*	B	26,2	13,1	38,4	4,9	23,2	29,2
			116* (MSH)	B	10,5	12,7	22,8	13,0	18,3	32,3
MSMEG_2784	msrB2	Methionine sulfoxide reductase	51*	B	14,2	27,0	37,7	2,2	36,3	38,5
MSMEG_3479	tpx	Thiol peroxidase	60* (MSH;Cys)	B	11,6	29,1	39,9	8,9	37,8	48,0
MSMEG_4085	MSMEG_4085	Nitrilotriacetate monooxygenase	336	B	31,2	14,3	32,2			
MSMEG_4309	ptpA	LMW protein-tyrosine-phosphatase	10*	B	22,2	11,2	30,6			
			58	E	41,1	47,7	79,9	10,2	17,3	31,5
**Transcription and Transcriptional regulators**										
MSMEG_0219	MSMEG_0219	RNA polymerase sigma factor	271	B	17,5	10,3	28,9	5,7	16,8	26,8
MSMEG_1367	rpoB	RNA polymerase beta SU	674	B	20,3	25,3	45,6	15,3	44,3	59,6
MSMEG_1368	rpoC	RNA polymerase beta’ SU	48*	B	19,6	14,3	29,5			
MSMEG_1515	MSMEG_1515	Two-component sensor histidine kinase	5	E	35,6	13,1	48,5	13,2	39,2	58,3
MSMEG_1831	whiB2	Transcriptional regulator WhiB2	67*	B	12,6	20,0	31,7			
			99*	B	10,5	33,6	44,9			
MSMEG_1874	mtrA	Two-component response regulator MtrA	68*	B	10,1	2,1	11,1			
MSMEG_1915	rshA	Anti-sigma-factor for SigmaH (RshA)	76*	B	38,2	15,8	53,2			
MSMEG_5071	rseA	Anti-sigma-factor for SigmaE (RseA)	67*	B	37,5	41,8	63,4	−3,4	49,1	45,7
MSMEG_2743	nrdR	Transcriptional repressor NrdR	71*	B	24,9	7,0	30,9			
MSMEG_4471	MSMEG_4471	MarR-family transcriptional regulator	58	B	42,3	12,3	54,0	34,6	25,8	61,1
MSMEG_4487	furB	Ferric uptake regulator FurB	124*	B	17,4	30,2	42,6	10,9	35,4	47,2
MSMEG_5768	MSMEG_5768	TetR family transcriptional regulator	61	E	22,7	12,5	22,7			
**Protein biosynthesis and quality control**										
MSMEG_1339	rpmG	50 S ribosomal protein L33-1	15*	B	23,9	30,5	53,4			
MSMEG_1468	rpsN	30 S ribosomal protein S14 type Z	27*	B	19,6	41,5	60,1	13,0	32,5	45,5
MSMEG_1520	rpmJ	50 S ribosomal protein L36	27*	B	33,5	21,7	43,6	13,5	22,4	35,9
MSMEG_1521	rpsM	30 S ribosomal protein S13	86* (MSH;Cys)	B	21,8	10,4	32,9	20,2	14,7	34,4
MSMEG_1579	rimI	Alanine acetyltransferase	55	B	38,4	9,7	51,0			
MSMEG_1878	MSMEG_1878	30 S ribosomal protein S30	83	E	40,4	46,7	84,6	22,7	59,7	82,6
MSMEG_2400	rpmB	50 S ribosomal protein L28	5*	B	35,7	40,3	74,5	26,5	41,8	70,4
			52	B	36,8	42,7	76,9	24,8	48,3	74,2
MSMEG_4951	rpmE	50 S ribosomal protein L31	16*	B	20,2	22,2	39,6	14,5	22,4	32,9
MSMEG_6895	rpsR2	30 S ribosomal protein S18-2	20*	B	24,6	73,6	84,8	8,9	76,0	84,9
			57* (MSH;Cys)	B	10,7	11,4	21,2	7,3	15,2	23,4
MSMEG_0839	lon1	ATP-dependent protease	72 (MSH)	B	25,7	11,1	38,5			
**Glycolysis/Gluconeogenesis and TCA cycle**										
MSMEG_0935	gpmA	2,3-bisphosphoglycerate-mutase	149	E	20,0	7,3	27,9	9,9	13,6	22,0
MSMEG_0970	MSMEG_0970	Phosphoglycerate mutase	146	B	10,6	13,4	21,5			
MSMEG_1547	pduC	Glycerol dehydratase large SU	156	B	12,2	20,6	31,0	8,3	37,5	46,5
			168	B	11,1	19,3	31,6	4,9	32,6	37,6
			342*	B	15,4	7,9	20,6	12,4	21,4	33,1
**Selected NaOCl-sensitive proteins with** >**10% increased thiol-oxidations under NaOCl stress in** ***M***. ***smegmatis***										
MSMEG_3227	pyk2	Pyruvate kinase	9*	B	10,6	10,1	20,6	8,7	21,2	30,4
MSMEG_5239	glpX	Fructose-1,6-bisphosphatase	205	B	12,5	13,7	23,9			
MSMEG_6759	glpK3	Glycerol kinase	294 (MSH)	B	10,7	8,9	16,8	9,9	19,9	29,8
MSMEG_0911	aceA	Isocitrate lyase	191	B	11,3	8,5	20,8	11,0	22,3	33,7
			268 (MSH)	B	11,4	12,7	20,3	12,8	24,8	37,6
MSMEG_1670	sdhA2	Succinate DH	385	B	15,5	31,0	35,1			
MSMEG_3640	glcB	Malate synthase G	612*	B	12,8	9,7	20,9			
MSMEG_4645	orB	a-OG ferredoxin oxidoreductase, beta SU	59*	B	10,1	9,4	20,9			
MSMEG_5676	citA	Citrate (Si) synthase	143* (**MSH;Cys**)	E	14,2	4,8	19,0	7,5	13,0	20,5
**Metabolism of Fatty acid and phospholipids**										
MSMEG_0913	umaA	Methoxy mycolic acid synthase 1	76* (**MSH**)	B	10,5	9,1	17,6			
MSMEG_1340	MSMEG_1340	(3 R)-hydroxyacyl-ACP dehydratase SU HadA	105	B	15,5	4,7	19,7	16,9	12,7	29,6
MSMEG_1342	MSMEG_1342	(3 R)-hydroxyacyl-ACP dehydratase SU HadC	127	B	14,0	8,7	22,0	2,8	24,5	39,7
MSMEG_1553	eutB	Ethanolamine ammonia-lyase	36	B	10,6	6,1	16,0	6,5	15,0	20,1
MSMEG_1554	eutC	Ethanolamine ammonia-lyase light chain	204*	B	11,7	15,7	26,4			
MSMEG_1807	accA3	Acetyl-/propionyl-CoA carboxylase alpha chain	236*	B	13,4	12,6	26,0	22,8	25,7	45,7
MSMEG_1813	accD5	Methylmalonyl-CoA carboxyltransferase	356 (MSH;Cys)	B	11,5	17,2	26,4	10,7	30,9	41,6
MSMEG_2207	MSMEG_2207	Beta-ketothiolase	9	B	12,9	11,0	26,2	−2,9	39,4	36,9
MSMEG_4116	MSMEG_4116	3-hydroxyacyl-CoA DH	148	B	18,8	12,9	34,4			
MSMEG_4327	kasA	3-oxoacyl-(Acyl-carrier-protein) synthase 1	171*	B	28,0	15,4	50,0			
MSMEG_4328	kasB2	3-oxoacyl-(Acyl-carrier-protein) synthase 1	227	B	26,0	11,7	36,0	12,2	21,6	34,9
MSMEG_4329	accD6	Acetyl/propionyl-CoA carboxylase (Beta SU)	191	B	19,5	14,2	32,7	14,6	28,2	38,4
			213	E	15,7	13,8	27,1			
			294 (MSH)	B	13,0	9,6	20,3			
MSMEG_4920	MSMEG_4920	Acetyl-CoA acetyltransferase	107*	B	43,0	0,6	43,3			
			398*	B	32,2	5,4	38,1			
MSMEG_5199	MSMEG_5199	Acetyl-CoA acetyltransferase	55	B	11,8	4,5	12,2			
MSMEG_5273	fadA3	Acetyl-CoA acetyltransferase	90*	B	20,0	8,7	28,0			
			390*	B	20,8	9,0	21,8			
MSMEG_5291	MSMEG_5291	Acyl-CoA synthase	16	B	17,1	4,5	22,5	20,2	12,0	32,2
			359	B	17,7	7,2	22,8	8,1	16,3	30,1
**Metabolism of nucleotides**										
MSMEG_1602	guaB	Inosine-5′-monophosphate DH	325* (**MSH**)	B	19,3	5,5	24,1	22,0	11,1	30,4
MSMEG_3634	guaB2	Inosine-5′-monophosphate DH	302* (**MSH**)	B	33,4	8,2	44,6	8,4	18,2	29,2
			321	B	15,5	12,6	32,0	7,8	12,5	20,5
**Metabolism of cofactors**										
MSMEG_0789	thiE	Thiamine-P synthase	20	B	24,1	5,1	28,2	18,0	19,5	37,9
MSMEG_0791	thiO	Glycine oxidase	32	B	44,3	1,2	42,5	26,1	9,5	36,3
MSMEG_0793	thiG	Thiazole synthase	75* (**MSH**)	B	10,3	9,1	17,6			
MSMEG_2671	folA	Dihydrofolate reductase	106	B	47,5	10,5	49,8	38,6	14,9	51,9
MSMEG_3067	ribD	Riboflavin biosynthesis protein RibD	78*	B	47,4	8,5	56,4			
MSMEG_3072	ribAB	Riboflavin biosynthesis protein RibBA	264*	B	19,3	52,3	65,2	7,3	61,7	69,1
MSMEG_3126	MSMEG_3126	Nitrogen fixation protein NifU	38*	B	18,1	22,4	38,9	7,3	32,8	41,7
MSMEG_4272	yfhF2	HesB/YadR/YfhF family protein	47* (**MSH**)	B	11,0	19,5	28,3	3,8	29,2	36,9
MSMEG_4827	MSMEG_4827	Acyl-CoA DH	44 (MSH)	E	32,5	35,4	66,7			
MSMEG_5698	moaA	Cyclic pyranopterin monoP synthase	50*	B	24,3	33,2	54,4	3,7	42,3	44,0
			305*	B	19,0	24,0	44,6			

Selected NaOCl-sensitive proteins with >10% increased thiol-oxidations in response to NaOCl stress in *M*. *smegmatis* as revealed using the OxICAT method. The *M*. *smegmatis* wild type and *mshC* mutant were harvested before (control) and 30 min after exposure to 1 and 0.5 mM NaOCl, respectively. Reduced and reversibly oxidized Cys residues were labelled with light and heavy ICAT, respectively, using OxICAT. Quantification of % thiol-oxidations was performed using MaxQuant software. The table includes MSMEG accessions, protein names, functions, surface accessabilities and % oxidation of Cys residues under control and NaOCl. (a) Conserved Cys are bold. (b) S-mycothiolated or S-cysteinylated Cys are marked with (+MSH) and (+Cys). (d) Relative surface accessibility (RSA) for buried (B) or exposed (E) Cys residues. (e) The % thiol-oxidation of each identified Cys peptide was calculated using MaxQuant. Based on the % thiol-oxidation of each Cys under control and NaOCl stress conditions, the % oxidation increase (% Diff NaOCl/co) was calculated under NaOCl-treatment and (f) average values are shown from at least three independent biological replicates. Selected NaOCl-sensitive peptides with >10% increased thiol-oxidation under NaOCl stress are shown here as a subset of the complete Tables S3–S4.

Under NaOCl stress, 381 Cys residues (34.7%) showed a >10% increased oxidation that include 116 Cys with >20% increased oxidations (Tables 2, S3 and S4, Fig. 5B,C). Among the NaOCl-sensitive proteins identified by OxICAT are 40 of 58 *S*-mycothiolated proteins that were found in the shotgun approach (Tables 1 and S1A). The overlap of S-mycothiolated proteins with NaOCl-sensitive proteins is further visualized in the *S*-mycothiolation treemap (Fig. 2), where *S*-mycothiolated proteins are color-coded according to their thiol-oxidations. Of note, most *S*-mycothiolated proteins with >10% increased oxidations under NaOCl stress represent abundant antioxidant and metabolic enzymes in the total proteome, such as Tpx, AhpC, Adh2, GlpK3, AceA, Kgd, Ino1, UmaA, AccD5 and IlvC as well as ribosomal proteins (RpsM, RplC) and RNA polymerase subunits (RpoC) (Fig. 2, Tables S1A, S3 and S4).

### NaOCl-sensitive proteins include antioxidant enzymes, Zn-containing alcohol dehydrogenases, ribosomal proteins and transcriptional regulators

Among the NaOCl-sensitive proteins are the *S*-mycothiolated peroxiredoxins Tpx and OsmC, the methionine-sulfoxide reductase MsrB2, the glutaredoxin NrdH and two catalases KatA2 and KatG3. The redox-sensitive low molecular weight protein-tyrosine-phosphatase PtpA showed 41.06% and 22.21% increased oxidation at Cys58 and in the Cys11-Cys15 active site motif, respectively (Tables 2, S3 and S4). PtpA was oxidized by H_2_O_2_ in its active site motif in *M*. *tuberculosis* which forms an intramolecular disulphide leading to enzyme inactivation^27^. In *M*. *tuberculosis*, S-nitrosylation at the non-conserved Cys53 was reported leading to a decreased enzymatic activity by interfering with protein stability and function^28, 29^.

Further highly oxidized Cys residues are the active site centres essential for catalysis or function in metal ion coordination (e.g. Zn, iron or FeS-clusters). These include proteins with structural or catalytic Zn-binding sites, such as Zn-finger motifs and Zn ribbons (Tables S3,S4, Fig. 5). Of note, 12 Zn-containing alcohol dehydrogenases, such as the abundant glycerol dehydrogenase MSMEG_6242 (Adh2) and 6 Adhs showed up-to 39% higher thiol-oxidation levels under NaOCl stress (AdhB1/B2/E1/E2, MSMEG_1138, MSMEG_1977 and MSMEG_4400). It is possible that these Adh enzymes participate in the glycerol oxidation pathway. These Zn-containing Adhs possess an N-terminal conserved catalytic Cys that was identified as NaOCl-sensitive in AdhE1 and MSMEG_1977. In addition, four conserved structural Cys residues are involved in Zn-binding (Figure S1D). Interestingly, AdhE1/E2/B2 and MSMEG_1138 are highly oxidized under NaOCl stress at the same Zn-binding Cys105, 106, 107 and 113, respectively. The inhibition of the yeast alcohol dehydrogenase YADH-1 due to overoxidation of its catalytic Cys has been shown previously^30^. Many ribosomal proteins with Zn-ribbon motifs showed >20% increased oxidation under NaOCl stress, including RpmG, RpmJ, RimL, RpmB, and RpsR2. RpsM is *S-*mycothiolated at the conserved Cys86 in *M*. *smegmatis* and *C*. *glutamicum* (Fig. S1E). Zn-containing ribosomal proteins are suggested to serve as reservoir for Zn-storage^31^.

Among the NaOCl-sensitive Zn-containing regulators are the Fur-family Zn-uptake regulator FurB (Zur), the NrdR repressor and the RshA and RseA anti-sigma factors. Zur has a CxxC Zn-redox switch motif that shows 17% increased oxidation under NaOCl stress. Zur is active as transcriptional repressor in the Zn-bound form, while Zn-deficiency leads to Zur inactivation and derepression of its regulon consisting of Zn-transporters, Zn-containing ribosomal proteins and the immunodominant ESAT-6 proteins^32^. The NrdR repressor is oxidized at the conserved Cys71 in its Zn ribbon motif. NrdR negatively regulates transcription of genes encoding two ribonucleotide reductases (*nrdF2* and *nrdF22*) and NrdH-like glutaredoxins (*MSMEG_1017* and *MSMEG_2297*) that are essential for *de novo* DNA synthesis^33^. Interestingly, the glutaredoxin NrdH is involved in reduction of NrdF and we identified both, the NrdR repressor and the glutaredoxin NrdH (MSMEG_2297) as NaOCl-sensitive proteins with >10% increased oxidations.

Two redox-regulatory anti-sigma factors RshA and RseA were identified as NaOCl-sensitive Zn-redox switches. RshA and RseA are ECF group-IV anti-sigma factors of the zinc-associated anti-sigma factor (ZAS) family (Figure S1F)^34^. RshA is oxidized at the conserved Cys76 with 38% increased oxidation under NaOCl stress. RseA showed 37% increased oxidation in its ZAS motif at Cys67 and Cys70 suggesting the formation of an intramolecular disulphide under NaOCl stress (Tables S3, S4, Fig. 5). The homolog of the RshA-SigH system of mycobacteria is the RsrA-SigR couple in *Streptomyces coelicolor*. Previous studies revealed that RsrA forms the disulphide between the N-terminal Cys11 and either Cys41 or Cys44 in the ZAS motif and the oxidized RsrA structure with the Cys11-Cys44 disulfide has been resolved recently^35, 36^. However, the N-terminal Cys11 of RsrA is not conserved in *M*. *smegmatis* RseA and we identified the redox switch in the ZAS motif of RseA as possible redox-signaling mechanism.

Using OxICAT, the unknown MarR family transcriptional regulator MSMEG_4471 was identified with 42% higher oxidation under NaOCl stress at Cys58 (Tables 2, S3, S4, Fig. 5). MSMEG_4471 is located adjacent to a multidrug-efflux transporter as possible target gene (MSMEG_4472) and has a homolog in *M*. *tuberculosis* (Rv2327) (Fig. S1G). MarR-family regulators often sense and respond to ROS and RES via conserved thiol-switch mechanisms (e.g. OhrR, SarZ, YodB, QsrR)^37, 38^. The function and redox-sensing mechanism of MSMEG_4471 and its related homolog of *M*. *tuberculosis* are subject of our current research.

We further identified many NaOCl-sensitive 4Fe4S-cluster-containing redox-switches, such as the dihydroxy-acid dehydratase IlvD, the molybdenum biosynthesis enzyme MoaA and the WhiB2 redox sensor (Fig. S1H). The FeS-cluster protein WhiB2 is essential in mycobacteria and required for septum formation and cell division^39^. In conclusion, Zn or FeS-cluster-binding thiol-containing proteins are often targets for oxidation under NaOCl stress and represent a large group in our list of NaOCl-sensitive proteins.

### NaOCl-sensitive proteins are involved in the central carbon metabolism and in the biosynthesis of fatty acids, cofactors, nucleotides and amino acids

Our OxICAT analysis identified 23 metabolic enzymes that are involved in energy metabolism with >10% increased oxidations under NaOCl stress (Tables 2, S3,S4, Fig. 5). The glycerol kinase GlpK3 and the large subunit of the glycerol dehydratase PduC showed 10% increased thiol-oxidations under NaOCl stress. Two NaOCl-sensitive phosphoglycerate mutases GpmA and MSMEG_0970 and the fructose-1,6-bisphosphatase GlpX are involved in the gluconeogenesis. The isocitrate lyase AceA and the malate synthase GlcB function in the glyoxylate cycle and are >10% oxidized under NaOCl-stress at conserved Cys residues. AceA was also *S-*mycothiolated at Cys268 and oxidation of GlcB was previously reported^40^. Other NaOCl-sensitive TCA cycle enzymes include the citrate synthase CitA that was identified as *S-*mycothiolated and *S-*cysteinylated at the conserved Cys143.

Furthermore, 27 NaOCl-sensitive enzymes function in the biosynthesis of fatty acids as precursors for mycolic acids. These include the mycothiolated acetyl-CoA carboxylases (AccD5 and AccD6) and the methoxy mycolic acid synthase (UmaA). Three acetyl-CoA acetyltransferases of the thiolase family MSMEG_4920, MSMEG_5199 and FadA3 showed >20% increased oxidations under NaOCl stress in their active sites that forms an acyl thioester intermediate during catalysis. In addition, enzymes required for the elongation of fatty acids were identified as NaOCl-sensitive, such as the 3-oxoacyl-ACP synthases KasA and KasB2 and the fatty acids synthase subunits HadA and HadC (FAS-I/II). Thus, the central carbon metabolism and the fatty acid biosynthesis pathways include many NaOCl-sensitive proteins that are important for cellular survival in mycobacteria.

We further identified 31 NaOCl-sensitive enzymes that function in cofactor biosynthesis, 11 nucleotide biosynthesis enzymes and 23 amino acid biosynthesis enzymes. Among the nucleotide biosynthesis enzymes are both *S*-mycothiolated IMP-dehydrogenases (GuaB and GuaB2) that showed >20% increased oxidation under NaOCl treatment at their conserved thioimidate active sites at Cys325 and Cys302, respectively.

In summary, using shotgun-LC-MS/MS and OxICAT analyses we identified 58 *S-*mycothiolated proteins and a >10% increased thiol-oxidation level for 33% of Cys residues under NaOCl stress that included also 40 *S*-mycothiolated Cys-peptides. The most interesting NaOCl-sensitive thiol-switches are the enzymes involved in energy metabolism, mycolic acid and fatty acid biosynthesis as well as the various Zn-containing alcohol dehydrogenases, ribosomal proteins and redox-sensing regulators, such as RseA, RshA, Zur and NrdR.

### Loss of mycothiol leads to 2-fold increased basal oxidations for 41% of all Cys residues in the *mshC* mutant redox proteome

**Figure 6:**
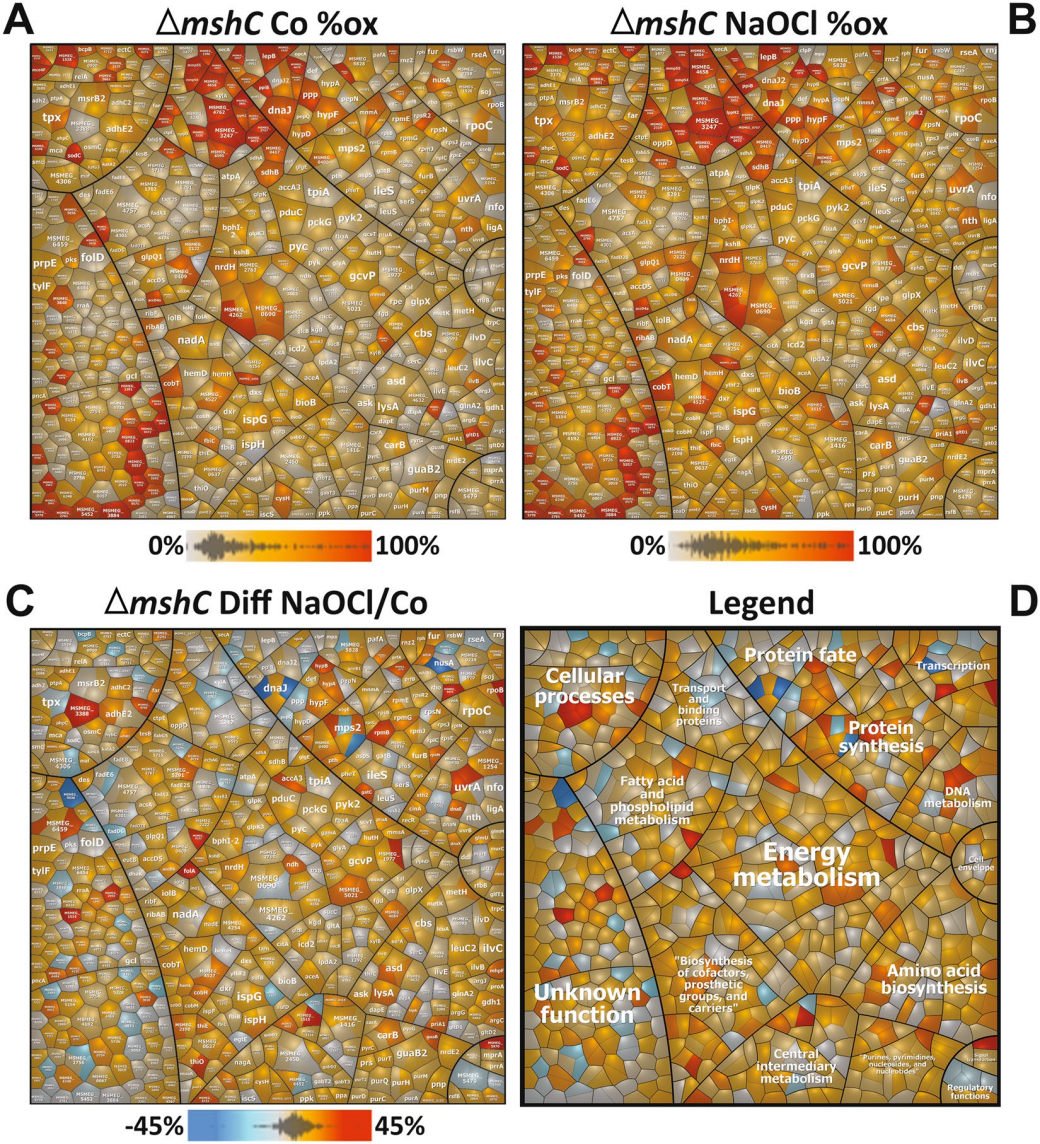
Voronoi redox treemaps show the percentages of thiol-oxidation levels of all Cys-peptides identified in the redox proteome of the *M. smegmatis mshC* mutant. The “*Voronoi redox treemaps*” show the percentages of thiol-oxidations of all 823 Cys-residues identified in the *mshC* mutant control (**A**) and 30 min after exposure to 0.5 mM NaOCl stress (**B**) The grey-yellow-red color gradient denotes 0–100% oxidation. The *Voronoi redox treemap* in (**C**) visualizes the percentages of oxidation changes under NaOCl stress using a blue-red color gradient ranging from −60 to +60% oxidation. The treemap in (**D**) is used as legend for the functional classification of the proteins displayed in (**C**). The treemaps are generated based on the OxICAT data presented in Table S4 using the Paver software (Decodon) and proteins were classified according to the *M*. *smegmatis* TIGRfam annotation.

Next, we investigated whether the loss of mycothiol affects the redox state in *M*. *smegmatis*. The redox state of 823 Cys residues was quantified using OxICAT in the *mshC* mutant under control and NaOCl stress as shown in the *Voronoi redox treemaps* (Fig. 6A–D, Tables S3, S4). Under control conditions, 485 Cys residues (58.9%) showed <25% oxidation levels, while 338 Cys residues (41.1%) were significantly oxidized with >25% increased oxidations. In contrast, only 21.8% oxidized thiols with >25% increased oxidation levels were quantified in the wild type. These results indicate that the content of reduced thiols is much lower in the *mshC* mutant compared to the wild type. Thus, the extent of reduced and oxidized thiols in the *mshC* mutant control resembles that of NaOCl-exposed wild-type cells as indicated in the pie chart diagram (Fig. 4). Furthermore, NaOCl stress resulted in >10% increased oxidations of 255 Cys residues in the *mshC* mutant. Thus, despite the higher basal level oxidation in the *mshC* mutant, the thiol-oxidation increase under NaOCl stress is comparable to that of the wild type with 34.7% and 30.8% oxidation increase in the wild type and *mshC* mutant, respectively (Fig. 4; Tables S3, S4).

To visualize the increased thiol-oxidation levels in the *mshC* mutant, the log2-fold-changes of the percentages in thiol-oxidations were calculated in the *mshC* mutant compared to the wild type (Figure S2; Tables S3, S4). It is interesting to note that especially NaOCl-sensitive and *S*-mycothiolated enzymes are higher oxidized in the *mshC* mutant and obviously vulnerable to oxidation in the absence of MSH. Proteins with 2–3 fold increased basal oxidation in the *mshC* mutant are involved in energy metabolism and fatty acid biosynthesis, such as TpiA, GlpK3, AceA, CitA, PckG, AccA3, AccD5, AccD6,MSMEG_0108, FadD6, FadE6, FadA3 and include many *S-*mycothiolated proteins. The mycothiolated Ino1 showed a 4-fold increased thiol-oxidation level at the conserved Cys18 in the NAD^+^ binding site in the *mshC* mutant. In addition, many enzymes that function in the biosyntheses pathways for cofactors and amino acids exhibit 2–3-fold increased oxidation levels in the *mshC* mutant.

Among the transcriptional regulators, we noticed 2–4-fold increased oxidation levels in the *mshC* mutant control for Cys residues of the RNA polymerase α, β and β’ subunits (RpoA/B/C), the transcription elongation and termination factors (NusA and Rho), the two-component sensor histidine kinase MSMEG_1515, the MarR-family regulator MSMEG_4471 and the FeS-cluster transcription factor WhiB1. WhiB1 is an essential DNA binding redox sensor with an NO-sensitive FeS-cluster that is nitrosylated and represses transcription of the essential chaperonin GroEL2 in *M*. *tuberculosis* under NO stress^41–43^. Earlier studies revealed also an interaction of WhiB1 with the alpha-1,4-glucan branching enzyme GlgB1 via the Cys residues that coordinate the FeS-cluster^44^. Interestingly, another FeS-cluster WhiB-family protein, WhiB3, is an important redox sensor of *M*. *tuberculosis* controlling EGT synthesis and thereby contributing to the redox and bioenergetics homeostasis^8^. Both, MSH and EGT are important for the redox balance, energy metabolism and virulence of *M*. *tuberculosis* ^8^. It is possible that WhiB-like proteins respond generally to hypochloric acid under infection conditions to module EGT biosynthesis, central carbon and fatty acid metabolism to promote intracellular survival.

### Most oxidized Cys residues in the OxICAT dataset are not surface-exposed

**Figure 7:**
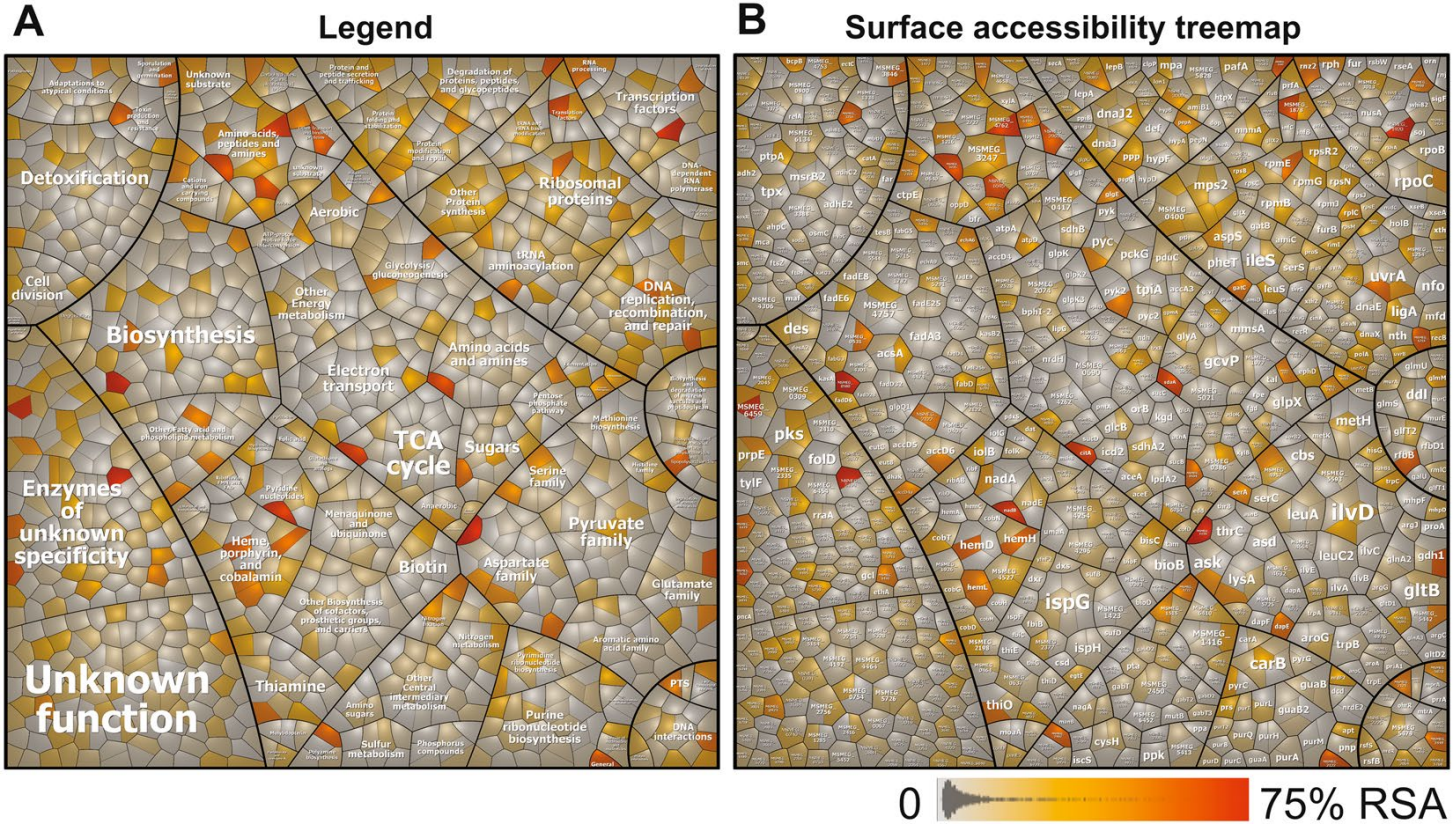
Relative surface accessibility (RSA) treemap of Cys residues identified in the redox proteome of *M. smegmatis* using OxICAT. The surface accessibilities of Cys residues were predicted using the NetSurfP server ver. 1.1 http://www.cbs.dtu.dk/services/NetSurfP/. The RSA treemap is composed of 1332 Cys residues identified in the redox proteome of the *M*. *smegmatis* wild type and the *mshC* mutant. Proteins are classified according to the *M*. *smegmatis* TIGRfam annotation. The treemap in (**A**) serves as legend for the functional classification of the proteins shown in the treemap in (**B**). The cells in the treemap represent Cys residues that are color-coded according to their relative surface accessibilities (RSA) with a white-orange-red color gradient ranging from 0–75% RSA. Exposed Cys are orange and buried Cys are shown in white-grey. In total, about 180 Cys residues (13.5%) in 163 proteins have a RSA value of >20% indicating that a majority of Cys residues are not surface-exposed in the predicted secondary structure of the proteins.

Next, we were interested if the Cys residues are oxidized since they are accessible and surface-exposed or if they are buried in the predicted secondary protein structure. We used the program NetSurfP^45^ (http://www.cbs.dtu.dk/services/NetSurfP/) to calculate the relative surface accessibilities (RSA) of all 1332 Cys residues identified in the *M*. *smegmatis* wild type and in the *mshC* mutant (Tables S3, S4). However, only 180 Cys residues (13.5%) have RSA values of >20% and are predicted as surface-exposed while most Cys residues are not predicted as surface-accessible. This is visualized in a *Surface accessibility treemap* where the RSA values of all Cys peptides are presented as white-red color gradient (Fig. 7). Moreover, among the 370 NaOCl-sensitive thiols, only 29 exposed Cys are predicted that include the *S-*mycothiolated proteins Rnz2, RpsR2, CitA, MSMEG_2799, LysA, AccD6 and MSMEG_4827. In contrast, highly redox-sensitive and nucleophilic active site Cys residues, such as Cys 302 and Cys325 of GuaB and GuaB2 are buried in the protein structure. Similarly, the *S-*mycothiolated active site Cys of the peroxiredoxins Tpx, AhpC and OsmC or the catalytic and Zn-binding sites of the Adhs are not surface-exposed, although these are major targets for NaOCl-induced oxidation. This indicates that the majority (86.5%) of the Cys residues in the *M*. *smegmatis* redox proteome are buried in the predicted secondary protein structure. These results are in agreement with previous predictions about the accessible surface area of Cys residues in the human, yeast and *E*. *coli* proteomes^46^.

### Thiol-oxidation of redox-sensitive regulators leads to increased transcription of the SigH, SigE, Zur and NrdR regulons in the transcriptome

The OxICAT results revealed an increased oxidation of many redox-sensitive transcriptional regulators under NaOCl stress, such as RseA, RshA, Zur and NrdR. Thiol-oxidation should lead to inactivation of these transcription factors resulting in transcriptional induction of the corresponding regulon members. To prove this hypothesis, we performed a RNA-seq transcriptome analysis of *M*. *smegmatis* after exposure to NaOCl stress. The m-value cut-off (log2-fold-change) for significant expression changes was defined as +/−4.47 (99% confidence, 3 bioreplicates, P < 0.001). In total, about 251 transcripts were significantly up-regulated under NaOCl stress in the transcriptome dataset including also the *sigH-rshA* operon (Table S5). This confirms previous transcriptome results in *C*. *glutamicum* where the SigH regulon was strongly induced under NaOCl stress^9^. The comprehensive SigH regulon has been recently defined in *M*. *tuberculosis* using ChIP-Seq analysis and 25 SigH-dependent promoter sequences were identified with the GGAAY-(N18/19)-GTT consensus^47^. Thus, we used this consensus to define the SigH regulon in *M*. *smegmatis* based on the RNA-seq data. First, we analyzed the upstream promoter sequences of the identified transcription start sites (TSS) among the NaOCl-induced transcriptional units for the SigH consensus using the MEME suite software. In total, 124 SigH-dependent promoter sequences were identified with the consensus sequence GGAAY-N18/19-GTT (p < 0.0001) and at least two-fold induction under NaOCl stress. SigH-dependent promoters were identified upstream upstream of 84 genes with m-values of ≥4.47 under NaOCl stress (Tables S6, S7). In total, 203 genes were identified that are transcribed from 124 SigH-dependent promoters either directly or as part of an operon.

Among these are 36 promoters that match the SigE promoter consensus GGAACY-N16/17-CGTT which could be recognized by both SigH and SigE-containing RNA polymerases^48^. The identified SigH/SigE-regulon members encode thioredoxins and thioredoxin reductases (Trx, Trx2, TrxB), chaperones and proteases (DnaK, DnaJ, GrpE, ClpB, Lon2, MSMEG_0424) and peroxiredoxins (AhpD). The comparison of the SigH-promoters identified in *M*. *tuberculosis* ^47^ with our list revealed only an overlap of 15 conserved SigH-promoter sequences. Thus, our *in silico* promoter analysis combined with NaOCl stress as inducing condition identified 109 new SigH-dependent promoters unique to *M*. *smegmatis*.

**Figure 8:**
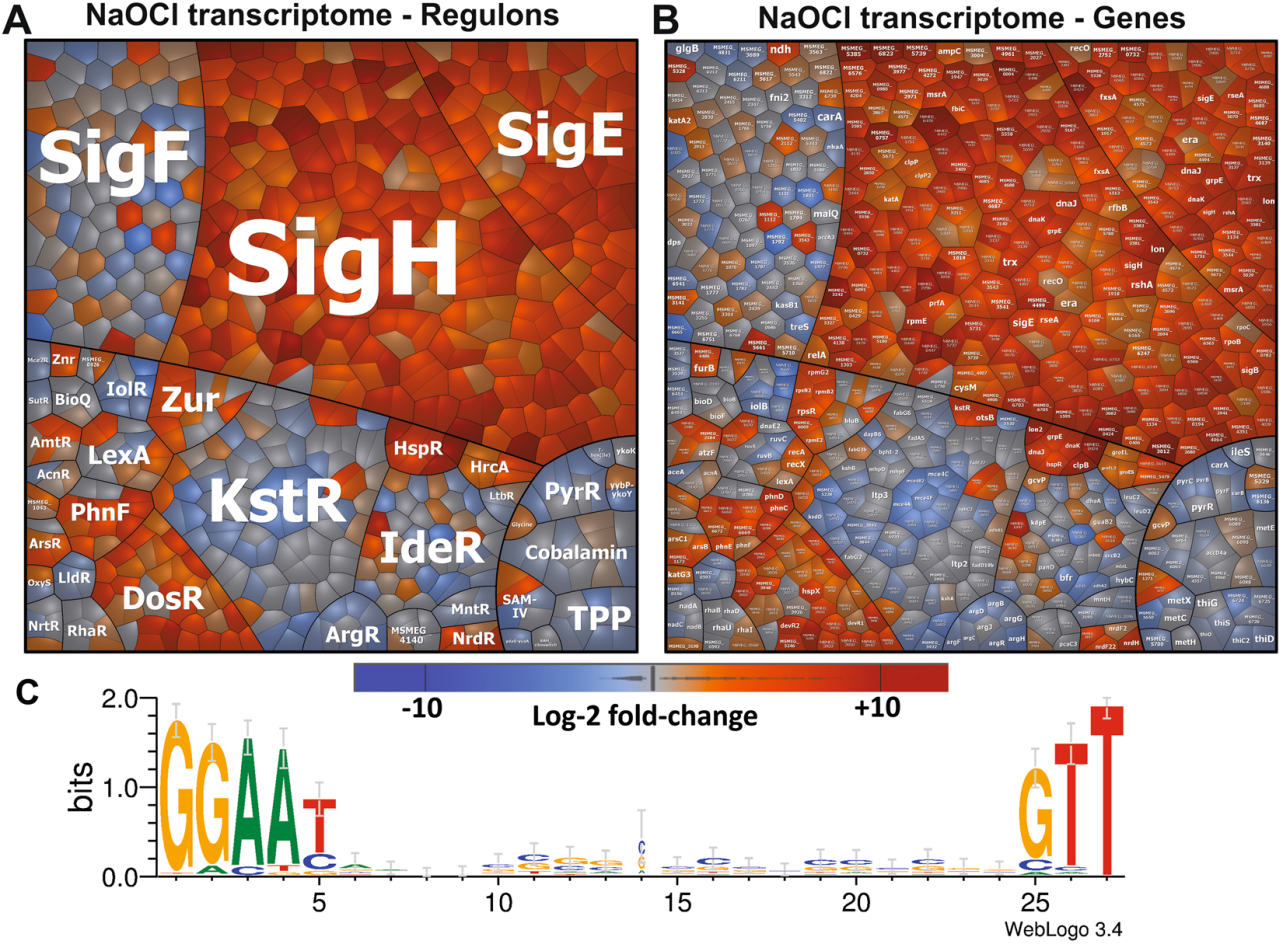
The transcriptome treemap indicates the strong induction of the SigH and SigE oxidative stress regulons under NaOCl stress in *M. smegmatis.* (**A**,**B**) The transcriptome treemap shows changes in gene expression of the *M*. *smegmatis* wild type under 1 mM NaOCl stress as log2-fold changes (m-values). The genes are classified into operons and regulons based on the RegPrecise database (http://regprecise.lbl.gov/RegPrecise/index.jsp) and the newly defined SigH and SigE regulons of this work. The regulon classification is used as legend (**A**) for the gene expression treemap in (**B**). Differential gene expression is visualized using an orange-blue color code where orange indicates log2-fold induction and blue repression of transcription under NaOCl stress. The oxidative stress-specific SigH and SigE-regulons are most strongly up-regulated under NaOCl stress. The Web-logo of the SigH-promoter consensus sequence (**C**) was created using WebLogo 3.0^78^ based on the alignment of 124 SigH-promoters identified in this work.

The gene expression data of the transcription factor regulons of *M*. *smegmatis* are visualized in a *Voronoi transcriptome treemap* using a blue-orange color gradient (Fig. 8). The SigH and SigE regulons represent the most strongly induced regulons under NaOCl treatment in this transcriptome treemap. In addition, expression of the NrdR regulon was increased under NaOCl stress, including the genes for the ribonucleotide reductases (*nrdF2* and *nrdF22*) and *nrdH* glutaredoxins (*MSMEG_1017* and *MSMEG_2297*). Thus, oxidation of NrdR leads to its inactivation and derepression of the NrdR regulon. In addition, the Zur repressor was identified as another NaOCl-sensitive Zn-redox-switch. Zur oxidation leads to derepression of the Zur regulons under NaOCl stress, including the *MSMEG_4486*-*zur* operon, genes for ribosomal proteins (*rpsR*, *rpsN2*, *rpmG2/B2/E2*) and zinc uptake transporters.

Transcription of the DosR dormancy regulon was elevated under NaOCl stress. The DosR regulon of *M*. *smegmatis* consists of *dosR* and a *dosR* paralog, universal stress proteins (MSMEG_5245, MSMEG_3945 and MSMEG_3950), a ribosome stabilizing-factor, diacylglycerol acyltransferases and nitroreductases and the [Ni-Fe]-hydrogenase Hyd3 (MSMEG_3931-3928). Hyd3 plays a role in hydrogen production under fermentation conditions to allow survival of *M*. *smegmatis* in oxygen-deprived environments^49^. The DosR regulon is induced in *M*. *tuberculosis* under dormany conditions by the gases NO, CO and hypoxia, which are sensed by the DosS and DosT heme sensor kinases^50^.

Furthermore, NaOCl stress leads to induction of two high-affinity phosphate uptake systems, the PstSCAB and the PhnDCE systems in the transcriptome. It has been shown in *E*. *coli* that NaOCl-treated cells encounter phosphate-starvation that is linked to the accumulation of polyphosphate as primordial chaperone in protection of proteins against NaOCl-induced unfolding^51, 52^. Thus, polyphosphate could also play an important role in the defense of mycobacteria against hypochloric acid under infection conditions which remains to be elucidated.

## Discussion

Protein *S*-mycothiolation is an emerging and widespread redox modification in Actinomycetes that particularly occurs under hypochlorite stress and functions in redox regulation and thiol-protection against overoxidation to sulphonic acids. Here, we aimed (i) to identify *S*-mycothiolated proteins using shotgun proteomics in *M*. *smegmatis*, (ii) to analyze the global thiol-oxidation state using OxICAT and (iii) to demonstrate the changes in gene expression due to thiol-oxidation in the RNA-seq transcriptome. We have selected NaOCl as infection-relevant condition for *S*-mycothiolation since our previous results revealed strongly increased *S*-thiolation in several bacteria under NaOCl^1^. Hypochloric acid (HOCl) is a strong thiol-oxidant with a high redox potential that targets Cys with a second-order rate constant of *k* = 3 × 10^7^ M^−1^ s^−1^ ^53^. The thiol group is first chlorinated to the unstable sulfenylchloride (-SCl) intermediate that reacts further with LMW thiols leading to *S*-thiolations, such as *S*-mycothiolations (-SSM) (Fig. 2)^53^. Protein *S*-mycothiolation was previously shown to function in thiol-protection and redox regulation in *C*. *glutamicum* under NaOCl stress.

Using the shotgun proteomics approach, we identified 58 *S-*mycothiolated proteins under NaOCl stress in *M*. *smegmatis* that participate in many essential cellular pathways, including energy metabolism, fatty acid and mycolic acid biosynthesis, nucleotide, cofactor and amino acid biosynthesis, redox regulation, transcription and translation to ensure survival and redox homeostasis under oxidative stress. Many *S-*mycothiolated proteins are conserved and essential targets for *S-*thiolation across Gram-positive bacteria, including the thiol-peroxidase Tpx, ribosomal proteins (RpsM), two IMP dehydrogenases (GuaB and GuaB2) and the myo-inositol-1-phosphate synthase (Ino1). Among the 58 targets for S-mycothiolation, 39 have Cys residues that are conserved also in the pathogen *M*. *tuberculosis* (Table S1A). Since these *S*-mycothiolated proteins were observed under infection-related conditions upon hypochlorite stress, they could be important to provide protection against the host immune defense in *M*. *tuberculosis*.

The quantitative redox proteomics approach OxICAT revealed an >10% increased thiol-oxidation level for 381 Cys residues (33.6%) under NaOCl stress. The 381 NaOCl-sensitive Cys-peptides overlap with 40 of 58 *S-*mycothiolated proteins which are also present at significant amounts in the proteome (Fig. 2). This indicates that protein *S*-mycothiolation is probably more abundant in *M*. *smegmatis* and that many more of the 381 NaOCl-sensitive proteins could represent *S*-mycothiolated proteins. However, due to the unstable nature of the MSH modification, the shotgun approach has limitations to detect only the tip-of-the-iceberg. Of note, 227 identified NaOCl-sensitive Cys residues are conserved in *M*. *tuberculosis* (Table S3) and could represent possible future drug-targets. This would be particularly attractive in combination with inhibitors of MSH biosynthesis to combat tuberculosis (TB) disease since MshB inhibitors are successfully applied in the clinical practice^54, 55^.

The conserved NaOCl-sensitive thiol-switches include Zn-containing proteins, such as alcohol dehydrogenases, ribosomal proteins, the ZAS anti sigma factors RseA and RshA and the transcriptional repressors Zur and NrdR. Using transcriptome analysis, we were able to demonstrate that thiol-oxidation leads to inactivation of NaOCl-sensitive transcriptional regulators resuling in changes of gene expression. Oxidation of both ZAS anti sigma factors (RshA and RseA) and the Zur and NrdR repressors was detected by OxICAT which is accompanied by the up-regulation of the corresponding regulons in the RNA-seq transcriptome. The oxidative stress responsive SigH and SigE regulons are major defense mechanisms and mediate redox homeostasis and protein quality control in mycobacteria and other actinomycetes^47, 48, 56, 57^. Moreover, based on the strong up-regulation of the SigH and SigE regulons in the transcriptome, we could identify 124 new SigH-dependent promoters that are transcribed under NaOCl stress and share the SigH promoter consensus sequence. Thus, our combined redox proteome and transcriptome results provide novel insights into the redox-signaling mechanisms of the RshA and RseA ZAS anti sigma factors and identified Zur and NrdR as new Zn-redox-sensors in mycobacteria. In *E. coli*, the chaperone holdase Hsp33 represents a NaOCl-sensitive Zn-redox switch that protects oxidatively damaged proteins against aggregation under oxidative stress^25, 58–61^. It will be interesting to elucidate if the newly identified conserved Zn-containing NaOCl-sensitive proteins function in protection against oxidative stress in *M. tuberculosis* during the infection cycle.

Our list of NaOCl-sensitive proteins includes many enzymes that have antioxidant functions or are involved in energy metabolism, such as glycerol catabolism and gluconeogenesis as well as in the biosynthesis pathways for fatty acids, mycolic acids, nucleotides and cofactors in *M*. *smegmatis* (Tables 2, S3 and S4). Some proteins that are susceptible to reversible thiol-oxidation by NaOCl stress in *M*. *smegmatis* were previously found as targets for *S*-nitrosylation in the *S*-nitrosoproteome of *M*. *tuberculosis* using the biotin-switch method^62, 63^. Of note, among the 29 *S*-nitrosylated proteins are also many antioxidant proteins and enzymes essential for the intermediary and fatty acid metabolism in *M*. *tuberculosis* ^63^. Specifically, 14 *S*-nitroslyated proteins overlap with the targets for protein thiol-oxidation in *M*. *smegmatis*, including the PEP carboxykinase PckG, the malate synthase GlcB and the citrate synthase AcnA and LpdA2, important for anaplerosis, gluconeogenesis and the TCA cycle. Common targets for thiol-oxidation and nitrosylation are further AtpA, SerA, PepN, GlnA2, FadD32, RpoB/C, KatG3 and Mpa. Using a transposon mutant screen, the proteasome was identified as major component in the defense against nitrosative stress including the ATPase Mpa that is required for NO-resistance and virulence in mice^64^. Another interesting target for *S*-nitrosylation and NaOCl-induced thiol-oxidation is the protein-tyrosine-phosphatase PtpA that is required for virulence in *M*. *tuberculosis* and was strongly oxidized in its active site motif under NaOCl stress^65^. This indicates that NO and NaOCl may target similar specific thiols in mycobacteria that functions in the protection against ROS and RNS to ensure cellular survival and virulence under infection conditions. In support of this notion, the transcriptome analysis revealed a strongly up-regulated DosR regulon under both, NaOCl and NO stress conditions suggesting that the DosS sensor kinase may function as redox sensor of NaOCl and NO^50, 66^. Thus, future studies should be directed to apply the NOxICAT and OxICAT approach^25, 26^ under NO and NaOCl stress in *M*. *tuberculosis* for more detailed comparison of the targets for nitrosylation and thiol-oxidation using similar methods.

The OxICAT data further revealed that the majority of NaOCl-sensitive thiols (24%) display only 10–20% increased thiol-oxidation levels under NaOCl stress. This is in agreement with the OxICAT data obtained in *E*. *coli* where only a subset of thiols was strongly oxidized under NaOCl treatment^25^. Using NetSurfP, we confirmed that only a minor part of 13.5% of all identified Cys residues are surface-exposed. The majority of NaOCl-sensitive thiols and *S-*mycothiolated proteins represent nucleophilic, catalytic or structural Cys residues that are buried and not solvent accessible in *M*. *smegmatis* further supporting that NaOCl targets specific redox-sensitive Cys residues with regulatory consequences resulting in changes of gene expression in the transcriptome.

To analyse the role of MSH for the thiol-oxidation state in *M*. *smegmatis*, we further analysed the changes in the thiol-redox proteome in the *mshC* mutant under NaOCl stress. Our results revealed that the absence of MSH leads to an increased basal level oxidation of 41.1% Cys residues that are >25% oxidized in the *mshC* mutant control compared to only 21.9% Cys residues that are >25% oxidized in the wild type control. NaOCl stress resulted in a further oxidation increase in the *mshC* mutant for 30.9% Cys residues that showed >10% oxidation increase. Thus, the level of 59% reduced and 41% oxidized thiols under control conditions in the *mshC* mutant resembled that of the wild type under NaOCl stress (Fig. 4). This difference in the thiol-redox state was even enhanced under NaOCl stress in the *mshC* mutant, with 34.4% reduced and 65.6% oxidized thiols. These results demonstrate in a quantitative manner the importance of MSH to maintain the reduced state of protein thiols and that protein thiols are more sensitive to NaOCl-induced oxidation in the absence of MSH.

The final question remains about the nature of the reversible thiol-modifications in the absence of MSH *in M*. *smegmatis*. Increased levels of EGT were previously reported in the *M*. *smegmatis mshA* mutant^67^. Both, MSH and EGT have been shown to be critical for redox homeostasis, energy metabolism and virulence in *M*. *tuberculosis* and mutants disrupted in MSH and EGT biosynthesis showed overlapping responses in the transcriptome^8, 15^. Thus, EGT might compensate for the absence of MSH and it will be subject of future studies to investigate whether the increased reversible thiol-oxidations in the *mshC* mutant represent *S*-ergothioneinylations.

## Methods

### Bacterial strains and growth conditions

*Mycobacterium smegmatis* mc^2^155 wild type and its isogenic ∆*mshC* mutant^68^ were cultivated in Hartmans-de Bont minimal medium (HdB) at 37 °C under vigorous agitation as described^16^. Cells were exposed to 0.5–1 mM NaOCl during the exponential growth phase at an optical density at 500 nm (OD_500_) of 0.4. Sodium hypochlorite (NaOCl) (15%) and N-ethylmaleimide (NEM) were purchased from Sigma Aldrich.

### Non-reducing MSH specific immunoblotting

About 25 µg of *M*. *smegmatis* protein extract was separated by non-reducing 12% SDS-PAGE and subjected to MSH specific immunoblot analysis using a polyclonal rabbit MSH antibody at dilution 1:1000 as described^9, 69^.

### Monobromobimane-labelling and HPLC-thiol metabolomics analysis

Thiol-labelling using monobromobimane (mBBr) was performed as described^10^. The mBBr-labelled thiols were separated by reverse phase chromatography and quantified by fluorescence detection using the same HPLC system as described^70^. The following gradient method was applied: 10 min 92% buffer A (10% methanol, 0.25% acetic acid, pH 3,9) supplemented with 8% buffer B (90% methanol, 0.25% acetic acid, pH 3,9), linear increase to 40% buffer B in 10 min, constant flow of 40% buffer B for 5 min, linear increase to 90% buffer B in 5 min, washing with 100% buffer B for 2 min followed by re-equilibration with 8% buffer B for 8 min. The flow rate was constantly set to 1.5 ml min^−1^.

### Identification of *S*-mycothiolated peptides using LTQ-Orbitrap Velos mass spectrometry

NEM-alkylated protein extracts from cells exposed to 1 mM NaOCl for 30 min were separated by 15% non-reducing SDS-PAGE followed by tryptic in-gel digestion and LTQ-Orbitrap-Velos mass spectrometry as described^9^. Post-translational thiol-modifications of proteins were identified by searching all MS/MS spectra in “dta” format against the *M*. *smegmatis* mc^2^155 target-decoy protein sequence database extracted from UniprotKB release 12.7 (UniProt Consortium, Nucleic acids research 2007, 35, D193-197) using Sorcerer™-SEQUEST^®^ (Sequest v. 2.7 rev. 11, Thermo Electron including Scaffold 4.0, Proteome Software Inc., Portland, OR). The SEQUEST search parameters and thiol-modifications were used as described^9^. The MS proteomics data (raw files and Scaffold files) are deposited into the ProteomeXchange database via the PRIDE partner repository with the dataset identifier PXD003303.

### Mass spectrometry (MS)-based thiol-redox proteomics using the OxICAT approach

To obtain 100 µg protein extract, 7–9 ml of the *M*. *smegmatis* wild type and *mshC* mutant cultures were harvested by centrifugation before and 30 min after treatment with 1 mM and 0.5 mM NaOCl, respectively. The OxICAT method was performed according to the protocol of Lindemann and Leichert^25, 26^ with the modification that cells were disrupted using a ribolyzer. The ICAT-labelled peptides were dissolved in 0.1% (v/v) acetic acid and loaded onto self-packed LC columns with 10 μl of buffer A (0.1% (v/v) acetic acid) at a constant pressure of 220 bar without trapping. Peptides were eluted using a non-linear 85 min gradient from 1 to 99% buffer B (0.1% (v/v) acetic acid in acetonitrile) with a constant flow rate of 300 nl/min and measured using Orbitrap mass spectrometry as described^71^.

### Quantification of thiol-oxidation using the MaxQuant software

The *M*. *smegmatis* mc^2^155 sequence database (accession CP000480 http://www.ncbi.nlm.nih.gov/nuccore/118168627) was used by the search engine Andromeda associated with the MaxQuant software (version 1.5.1.2) to quantify the ICAT-labelled Cys peptides. Two miscleavages were allowed, the parent ion mass tolerance was 10 ppm and the fragment ion mass tolerance was 1.00 Da. The average percentage of oxidation of each Cys peptide and the percentage change under NaOCl stress were calculated from three independent biological replicates using the intensity values provided by MaxQuant. Voronoi treemaps were generated using the Paver software to visualize the percentage oxidation of all identified ICAT-labelled peptide pairs. The MS raw files and MaxQuant search files are deposited into the ProteomeXchange database via the PRIDE partner repository with the dataset identifier PXD003303.

### RNA isolation, library preparation and next generation cDNA sequencing

*M*. *smegmatis* mc^2^155 wild-type cells were grown in 3 biological replicates, harvested before and 30 min after exposure to 1 mM NaOCl stress and disrupted in RNA lysis buffer containing 3 mM EDTA and 200 mM NaCl with a Precellys24 Ribolyzer. RNA isolation was performed using the acid phenol extraction protocol as described^9^. The RNA quality was checked by Trinean Xpose (Gentbrugge,Belgium) and the Agilent RNA Nano 6000 kit using an Agilent 2100 Bioanalyzer (Agilent Technologies, Böblingen, Germany). Ribo-Zero rRNA Removal Kit (Bacteria) from Illumina (San Diego, CA, USA) was used to remove the rRNA. TruSeq Stranded mRNA Library Prep Kit from Illumina (San Diego, CA, USA) was used to prepare the cDNA libraries. The resulting cDNAs were sequenced paired end on an Illumina HiSeq 1500 and MiSeq system (San Diego, CA, USA) using 75 bp read length. The transcriptome sequencing raw datafiles are available in the ArrayExpress database (www.ebi.ac.uk/arrayexpress) under accession number: E-MTAB-4522.

### Bioinformatics data analysis, read mapping, data visualization and analysis of differential gene expression

Trimmed reads (26 nt) were mapped to the *M*. *smegmatis* mc^2^155 genome sequence (accession number NC_008596) using SARUMAN^72^, allowing one error per read. The forward and reverse reads, if both present and with a maximum distance of 1 kb, were combined to one read containing the reference sequence as insert. Paired mappings with a distance >1 kb were discarded, and paired reads with either only the forward or the reverse read mapping were retained as single mapping reads. For the visualization and counting of short read alignments, ReadXplorer v2.2^73^ was used.

Differential gene expression analysis was performed using the software DEseq2^74^ included in the ReadXplorer v2.2 software^73^. The signal intensity value (a-value) was calculated by log2 mean of normalized read counts and the signal intensity ratio (m-value) by log2 fold change. The evaluation of the differential RNAseq data was performed using an adjusted p-value cut-off of P ≤ 0.01 and a signal intensity ratio (m-value) cut-off of ≥4.47 or ≤−4.47. The latter was determined by applying a significance level of 1% to the experiment with the assumption that the majority of genes are not differentially transcribed. Thus, 99% of all m-values should fall within this range. Therefore, the standard deviation (STDEV) for all m-values was calculated and the cut-off was set to m = 2.58 * STDEV. Genes with an m-value outside this interval and P ≤ 0.01 were considered as differentially transcribed. For the identification of operons, ReadXplorer v2.2 was used^73^. Therefore, all mapped reads were combined and if two neighbouring genes were connected by at least 20 spanning reads, the genes were considered as an operon.

### Identification of SigH- and SigE-regulated promoters with increased transcription under NaOCl stress

The upstream regions of 25 genes with the highest induction under NaOCl stress were selected for manual prediction of promoters. In case of accumulation of mapped reads as visualized by ReadXplorer v2.2, the region −5 nt to −55 nt was defined as promoter region^73^. For 20 genes, a stack of mapped reads and the corresponding promoter could be identified. These identified promoter regions were analyzed using the MEME algorithm^75^ and the MEME Suite^76^ to identify conserved promoter sequences. The identified conserved motif showed high similarity to SigH/SigE-dependent −35 (GGAAY) and −10 (GTT) promoter motifs separated by 17 or 18 bases^48^. The original MEME motif and an artificial MEME motif with a spacer length of 18 bases were used in the search for additional SigH-dependent promoter sequences. The 5′-regions (−300 nt–+100 nt) of all genes with 2-fold higher expressions after NaOCl treatment (m-value ≥ 1) (P ≤ 0.01) were scanned for both MEME motifs using FIMO^77^ with a p-value < 0.0001 of the MEME Suite software^76^. The identified promoter motifs were manually checked for the presence of the highly conserved SigH/SigE-dependent −35 (GGAAY) and −10 (GTT) promoter motifs^48^. One mismatch was allowed at position 1 or 2 of the −35 motif and another one within the −10 motif. Finally, the clear accumulation of mapped reads downstream of the identified promoters was validated using ReadXplorer v2.2^73^.

## Electronic supplementary material

Supplementary Figures and Tables

Supplementary Table S1A

Supplementary Table S1B

Supplementary Table S1C

Supplementary Table S1D

Supplementary Table S2

Supplementary Table S3

Supplementary Table S4

Supplementary Table S5

Supplementary Table S6

Supplementary Table S7
