# Supplementary material for: Monitoring global protein thiol-oxidation and protein S-mycothiolation in Mycobacterium smegmatis under hypochlorite stress
Source: Sci Rep. 2017 Apr 26;7:1195. doi: 10.1038/s41598-017-01179-4 (PMC5430705; doi:10.1038/s41598-017-01179-4)
Supplement: Supplementary file 1 — Supplementary Figures and Tables [file 41598_2017_1179_MOESM1_ESM.pdf]

Supplementary Information for

Monitoring global protein thiol-oxidation and *S*-mycothiolation in *Mycobacterium smegmatis* under hypochlorite stress

Melanie Hillion[1], Jörg Bernhardt[2], Tobias Busche[3], Martina Rossius[1], Sandra Maaß[2], Dörte Becher[2], Mamta Rawat[4], Markus Wirtz[5], Rüdiger Hell[5], Christian Rückert[3,6], Jörn Kalinowski[3] and Haike Antelmann[1]*

Departments & Institutions:

[1]*Institute for Biology-Microbiology, Freie Universität Berlin, D-14195 Berlin, Germany;*

[2]*Institute for Microbiology, Ernst-Moritz-Arndt-University of Greifswald, D-17487 Greifswald, Germany;*

[3]*Center for Biotechnology, Bielefeld University, D-33594 Bielefeld, Germany*

[4]*Department of Biology, California State University-Fresno, Fresno, CA 937401, United States*

[5]*Plant Molecular Biology, Centre for Organismal Studies Heidelberg, University of Heidelberg, Heidelberg, Germany*

[6]*Sinskey Lab, Department of Biology, Massachusetts Institute of Technology, Cambridge, MA, USA*

Running title: Protein thiol-oxidation and protein *S-mycothiolation* in *Mycobacterium smegmatis*

***Corresponding author:**

Haike Antelmann,Institute for Biology-Microbiology, Freie Universität Berlin,
D-14195 Berlin, Germany,Tel.+49-30-838-51221, Fax.+49-30-838-451221,

E-mail: haike.antelmann@fu-berlin.de

Supplementary Figure legends

Figure S1. **ClustalΩ sequence alignments of NaOCl-sensitive thiol-switches with increased thiol-oxidation and/or *S*-mycothiolations in NaOCl-treated *M. smegmatis* cells.** The alignments include redox-sensitive transcriptional regulators (RseA, RshA, IdeR, MSMEG_4471, WhiB1/2), metabolic enzymes and ribosomal proteins, such as the myoinositol-phosphate synthase (Ino1), the IMP dehydrogenases (GuaB and GuaB2), the ribosomal protein (RpsM) and Zn-containing alcohol dehydrogenases (AdhB1/B2/E1/E2/C2/MSMEG_1138, MSMEG_3464, MSMEG_4400, MSMEG_1977). The alignments were performed using ClustalΩ (http://www.ebi.ac.uk/Tools/msa/clustalo/) and presented using Jalview (ver.2.9.0.b2). The purple color gradient indicates percentage of amino acid identity. Cysteine residues are marked in red and the oxidized Cys are bold-faced and marked with an asterisk (*). The *S*-mycothiolated Cys is marked with "[M]".

(A) The iron-dependent repressor IdeR of *M. smegmatis* was aligned with *M. tuberculosis* IdeR (Rv2711) and DtxR from *C. glutamicum* (Cg2103) and *C. diphtheriae* (DIP1414). The conserved single Cys102 is *S*-mycothiolated in *M. smegmatis* after NaOCl stress.

(B) The inositol-3-phosphate synthase (Ino1) of *M. smegmatis* was aligned with its homologs of *M. tuberculosis* (Rv0046c), *C. glutamicum* (Cgl2996) and *C. diphtheriae* (DIP0115). The conserved Cys18 was *S*-mycothiolated in *M. smegmatis* with a 6.3% oxidation increase under NaOCl stress. Ino1 of *C. glutamicum* was *S*-mycothiolated at Cys79.

(C) The inosine-5'-monophosphate dehydrogenases GuaB and GuaB2 of *M. smegmatis* were aligned with the GuaB homologs of *M. tuberculosis* and *C. glutamicum*. The active site Cys325 of GuaB and Cys302 of GuaB2 were identified as *S*-mycothiolated and with 19% and 33% increased oxidations, respectively, under NaOCl stress. This Cys form a thioimidate intermediate during the catalysis and is one of the most conserved NaOCl-sensitive targets for *S*-thiolation across different bacteria.

(D) The Zn-containing alcohol dehydrogenases of *M. smegmatis* AdhB1/B2/E1/E2/C2, MSMEG_3464, MSMEG_4400, MSMEG_1977 were aligned. Two catalytic Cys residues are

shown in red and the four Zn-binding Cys residues are labelled in blue. Interestingly, AdhE1 and MSMEG_1977 were oxidized at their catalytic N-terminal Cys58 and Cys39, while AdhB1/B2/E2 and MSMEG_1138 were all oxidized at one conserved Zn-binding Cys105, 106, 107 and 113.

(E) The ribosomal protein RpsM of *M. smegmatis* (MSMEG_1521) was aligned with RpsM homologs of *M. tuberculosis* (Rv3460c), *C. glutamicum* (Cg0673) and *C. diphtheriae* (DIP0546). The single Cys86 is conserved and *S*-mycothiolated in both *M. smegmatis* and *C. glutamicum*, as well as *S*-cysteinylated in *M. smegmatis* and *C. glutamicum mshC* mutants. Cys86 showed a 21.8% oxidation increase in the *M. smegmatis* wild type under NaOCl stress.

(F) The anti-sigma factors RshA (MSMEG_1915) and RseA (MSMEG_5071) of *M. smegmatis* were aligned with their homologs of *M. tuberculosis* (Rv3221A and Rv1222 respectively) and *C. glutamicum* (Cg0877 and Cg1272). RseA showed 37.5% increased oxidation in the Zn-binding (ZAS) motif at Cys67 and Cys70 and RshA was 38.2% higher oxidized at the conserved Cys76 not present in the ZAS motif.

(G) The MarR-type transcriptional regulator MSMEG_4471 of *M. smegmatis* was aligned with homologs of *M. tuberculosis* (Rv2327), *Rhodococcus equi* (REQ_39840) and *Nocardia cyriacigeorgica* (NOCYR_0508). Cys58 showed an 42.3 % oxidation increase in *M. smegmatis* wild-type strain under NaOCl stress.

(H) WhiB1 and WhiB2 of *M. smegmatis* (MSMEG_1919 and MSMEG_1831) were aligned with their homologs of *M. tuberculosis* (Rv3219 and Rv3260c) and *C. glutamicum* (Cg0878 and Cg0850). Conserved Cys residues of WhiB1 and WhiB2 coordinate an [4Fe-4S] cluster. The conserved Cys9 of WhiB1 and Cys67 of WhiB2 showed increased oxidation of 7.4% and 12.6%, respectively, under NaOCl stress.

Fig. S1A

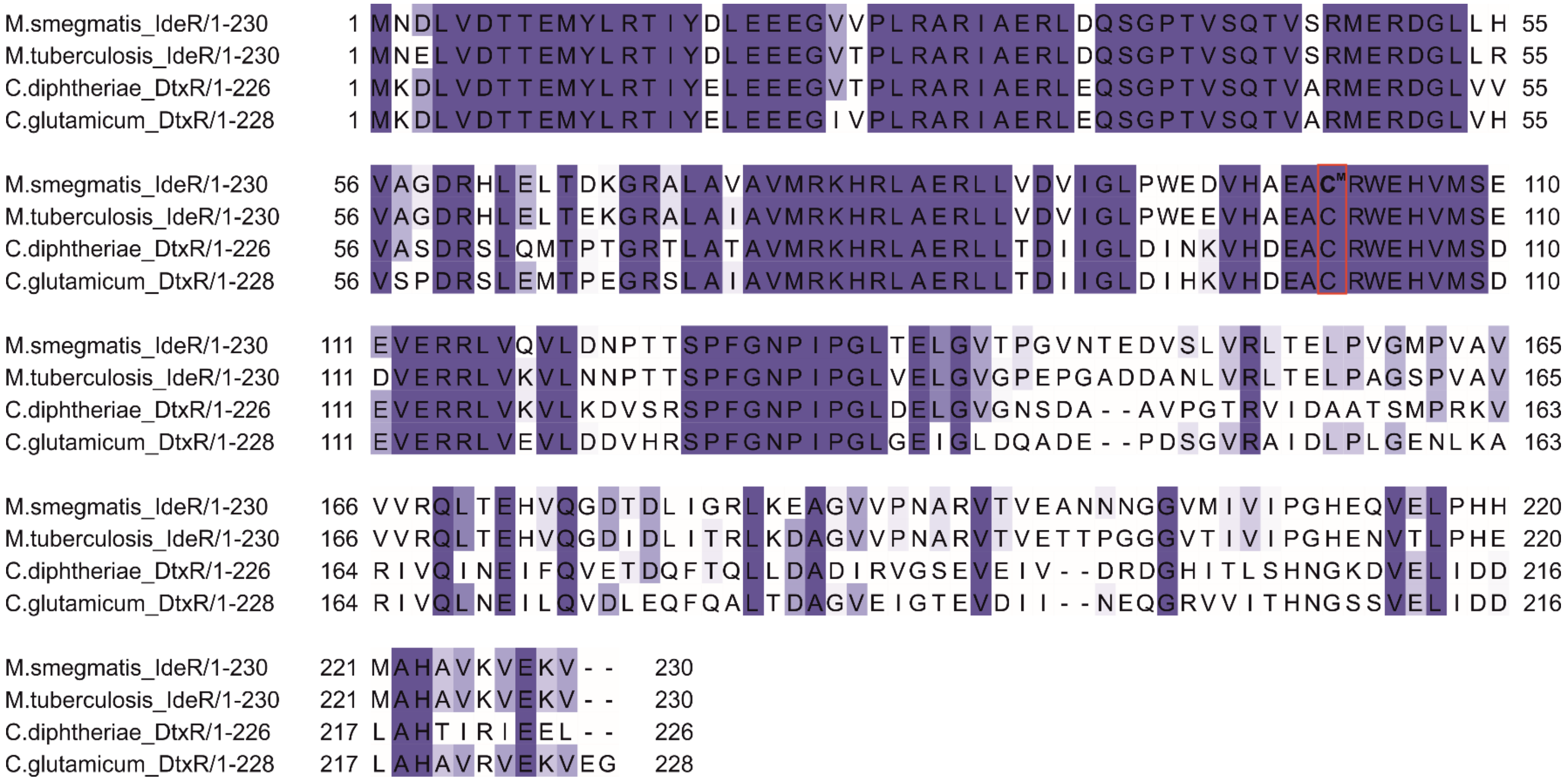

M.smegmatis_IdeR/1-230 1 MNDLVDTTEMYLRTIYDLEEEGVVPLRARIAERLDQSGPTVSQTVSRMERDGLLH 55
M.tuberculosis_IdeR/1-230 1 MNELVDTTEMYLRTIYDLEEEGVTPLRARIAERLDQSGPTVSQTVSRMERDGLLR 55
C.diphtheriae_DtxR/1-226 1 MKDLVDTTEMYLRTIYELEEEGVTPLRARIAERLEQSGPTVSQTVARMERDGLVV 55
C.glutamicum_DtxR/1-228 1 MKDLVDTTEMYLRTIYELEEEGIVPLRARIAERLEQSGPTVSQTVARMERDGLVH 55
M.smegmatis_IdeR/1-230 56 VAGDRHLELTDKGRALAVAVMRKHRLAERLLVDVIGLPWEDVHAEACRWEHVMSE 110
M.tuberculosis_IdeR/1-230 56 VAGDRHLELTEKGRALAIAVMRKHRLAERLLVDVIGLPWEEVHAEACRWEHVMSE 110
C.diphtheriae_DtxR/1-226 56 VASDRSLQMTPTGRTLATAVMRKHRLAERLLTDIIGLDINKVHDEACRWEHVMSD 110
C.glutamicum_DtxR/1-228 56 VSPDRSLEMTPEGRSLAIAVMRKHRLAERLLTDIIGLDIHKVHDEACRWEHVMSD 110
M.smegmatis_IdeR/1-230 111 EVERRLVQVLDNPTTSPFGNPIPGLTELGVTPGVNTEDVSLVRLTELPVGMPVAV 165
M.tuberculosis_IdeR/1-230 111 DVERRLVKVLNNPTTSPFGNPIPGLVELGVGPEPGADDANLVRLTELPAGSPVAV 165
C.diphtheriae_DtxR/1-226 111 EVERRLVKVLKDVSRSPFGNPIPGLDELGVGNSDA--AVPGTRVIDAATSMPRKV 163
C.glutamicum_DtxR/1-228 111 EVERRLVEVLDDVHRSPFGNPIPGLGEIGLDQADE--PDSGVRAIDLPLGENLKA 163
M.smegmatis_IdeR/1-230 166 VVRQLTEHVQGDTDLIGRLKEAGVVPNARVTVEANNNGGVMIVIPGHEQVELPHH 220
M.tuberculosis_IdeR/1-230 166 VVRQLTEHVQGDIDLITRLKDAGVVPNARVTVETTPGGGVTIVIPGHENVTLPHE 220
C.diphtheriae_DtxR/1-226 164 RIVQINEIFQVETDQFTQLLDADIRVGSEVEIV--DRDGHITLSHNGKDVELIDD 216
C.glutamicum_DtxR/1-228 164 RIVQLNEILQVDLEQFQALTDAGVEIGTEVDII--NEQGRVVITHNGSSVELIDD 216
M.smegmatis_IdeR/1-230 221 MAHAVKVEKV-- 230
M.tuberculosis_IdeR/1-230 221 MAHAVKVEKV-- 230
C.diphtheriae_DtxR/1-226 217 LAHTIRIEEL-- 226
C.glutamicum_DtxR/1-228 217 LAHAVRVEKVEG 228

Fig. S1B

```
M.smegmatis_Ino1/1-363        1 --------MSEHAGEIRVAIVGVGNC*ASSLVQGVQYYRNADENTTVPGLMHVKFG 47
M.tuberculosis_Ino1/1-367     1 MSEHQSLPAPEASTEVRVAIVGVGNCASSLVQGVEYYYNADDTSTVPGLMHVRFG 55
C.diphtheriae_DIP0115/1-355   1 ------------MSAIRVAIAGVGNCASSLVQGVEYYKDASPDQQVPGLMHVQFG 43
C.glutamicum_Cgl2996/1-363    1 ----------MSTSTIRVAIAGVGNCATSLIQGVEYYRNADPSETVPGLMHVKFG 45

M.smegmatis_Ino1/1-363       48 PYHVRDVNFVAAFDVDAKKVGFDLSEAIFASENNTIKIADVPPTDVIVQRGPTLD 102
M.tuberculosis_Ino1/1-367    56 PYHVRDVKFVAAFDVDAKKVGFDLSDAIFASENNTIKIADVAPTNVIVQRGPTLD 110
C.diphtheriae_DIP0115/1-355  44 DYHVGDIEFVAAFDVDRAKVGLDLSEAINASENCTIRICDVPETGVTVQRGPTLD 98
C.glutamicum_Cgl2996/1-363   46 DYHVGDIEFVAAFDVDAEKVGIDLADATEASQNCTIKIADVPQTGINVLRGPTLD 100

M.smegmatis_Ino1/1-363      103 GIGKYYADTIEVSDAEPVDVVKVLKEAEVDVLVSYLPVGSEEADKFYAQCAIDAG 157
M.tuberculosis_Ino1/1-367   111 GIGKYYADTIELSDAEPVDVVQALKEAKVDVLVSYLPVGSEEADKFYAQCAIDAG 165
C.diphtheriae_DIP0115/1-355  99 GLGKYYRATVEESPAQAVDVVQVLKDERVDVLVSYLPVGSEEADKFYAQCAIDAN 153
C.glutamicum_Cgl2996/1-363  101 GLGDHYRATIDESTAEPVDVVQALIDAKADVLVSYLPVGSEEADKFYAQAAIDAG 155

M.smegmatis_Ino1/1-363      158 VAFVNALPVFIASDPVWAKKFEDAGVPIVGDDIKSQVGATITHRVMAKLFEDRGV 212
M.tuberculosis_Ino1/1-367   166 VAFVNALPVFIASDPVWAKKFTDARVPIVGDDIKSQVGATITHRVLAKLFEDRGV 220
C.diphtheriae_DIP0115/1-355 154 VAFVNALPVFIASDPQWAAKFEEAGVPIVGDDIKSQVGATITHRVLAKLFEDRGV 208
C.glutamicum_Cgl2996/1-363  156 CAFVNALPVFIASDPEWAKKFTDAGIPIVGDDIKSQIGATITHRVLARLFEERGV 210

M.smegmatis_Ino1/1-363      213 TLDRTYQLNVGGNMDFLNMLERSRLESKKVSKTQAVTSNLS-GALAGKVEDKNVH 266
M.tuberculosis_Ino1/1-367   221 QLDRTMQLNVGGNMDFLNMLERERLESKKISKTQAVTSNLK-R----EFKTKDVH 270
C.diphtheriae_DIP0115/1-355 209 HLDRTMQLNVGGNMDFKNMLERERLESKKISKTQAVTSNLD-Q----EIAARDVH 258
C.glutamicum_Cgl2996/1-363  211 RVDRTMQLNVGGNMDFKNMLDRNRLESKKVSKTQAVTSNIPDGPLSGKVEDRNVH 265

M.smegmatis_Ino1/1-363      267 IGPSDHVAWLDDRKWAYVRLEGRAFGDVPLNLEYKLEVWDSPNSAGVIIDAVRAA 321
M.tuberculosis_Ino1/1-367   271 IGPSDHVGWLDDRKWAYVRLEGRAFGDVPLNLEYKLEVWDSPNSAGVIIDAVRAA 325
C.diphtheriae_DIP0115/1-355 259 IGPSDYVGWLDDRKWAYVRLEGTAFGDVPLNLEYKLEVWDSPNSAGIIIDALRAA 313
C.glutamicum_Cgl2996/1-363  266 IGPSDHVQWLDDRKWAYVRLEGTAFGGVPLNLEYKLEVWDSPNSAGIIIDAVRAA 320

M.smegmatis_Ino1/1-363      322 KIAKDRGIGGPIEAASAYLMKSPPKQLADDVARAELETFIEG-  363
M.tuberculosis_Ino1/1-367   326 KIAKDRGIGGPVIPASAYLMKSPPEQLPDDIARAQLEEFIIG-  367
C.diphtheriae_DIP0115/1-355 314 KIAKDRGIGGPVYPAAAYLMKSPPRQMRDEAARAELEQFISG-  355
C.glutamicum_Cgl2996/1-363  321 KIALDRGIGGPIMPASSYLMKSPPEQLPDDVARERLEAFIIEA  363
```

Fig. S1C

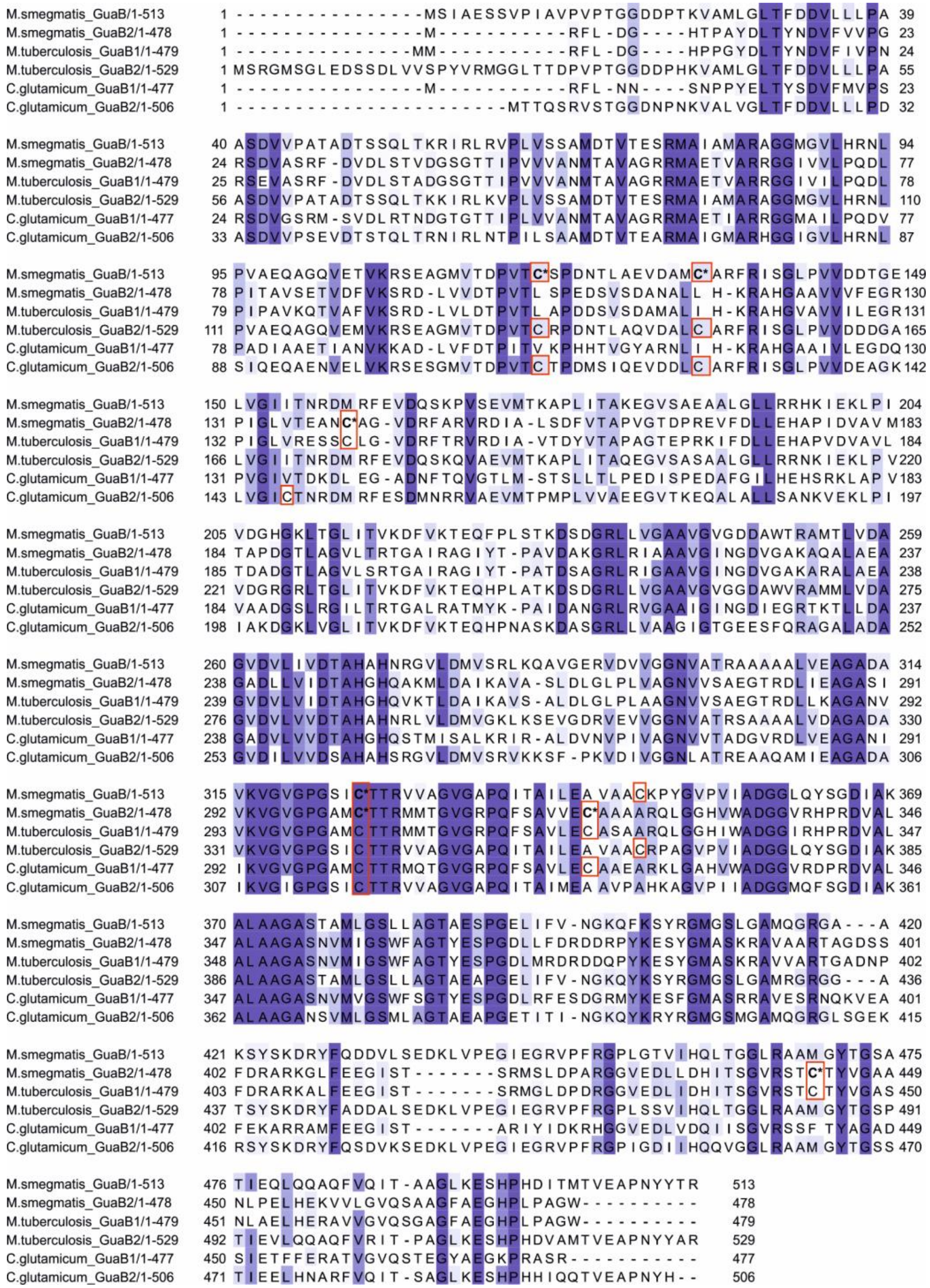
M.smegmatis_GuaB/1-513
M.smegmatis_GuaB2/1-478
M.tuberculosis_GuaB1/1-479
M.tuberculosis_GuaB2/1-529
C.glutamicum_GuaB1/1-477
C.glutamicum_GuaB2/1-506

Fig. S1D

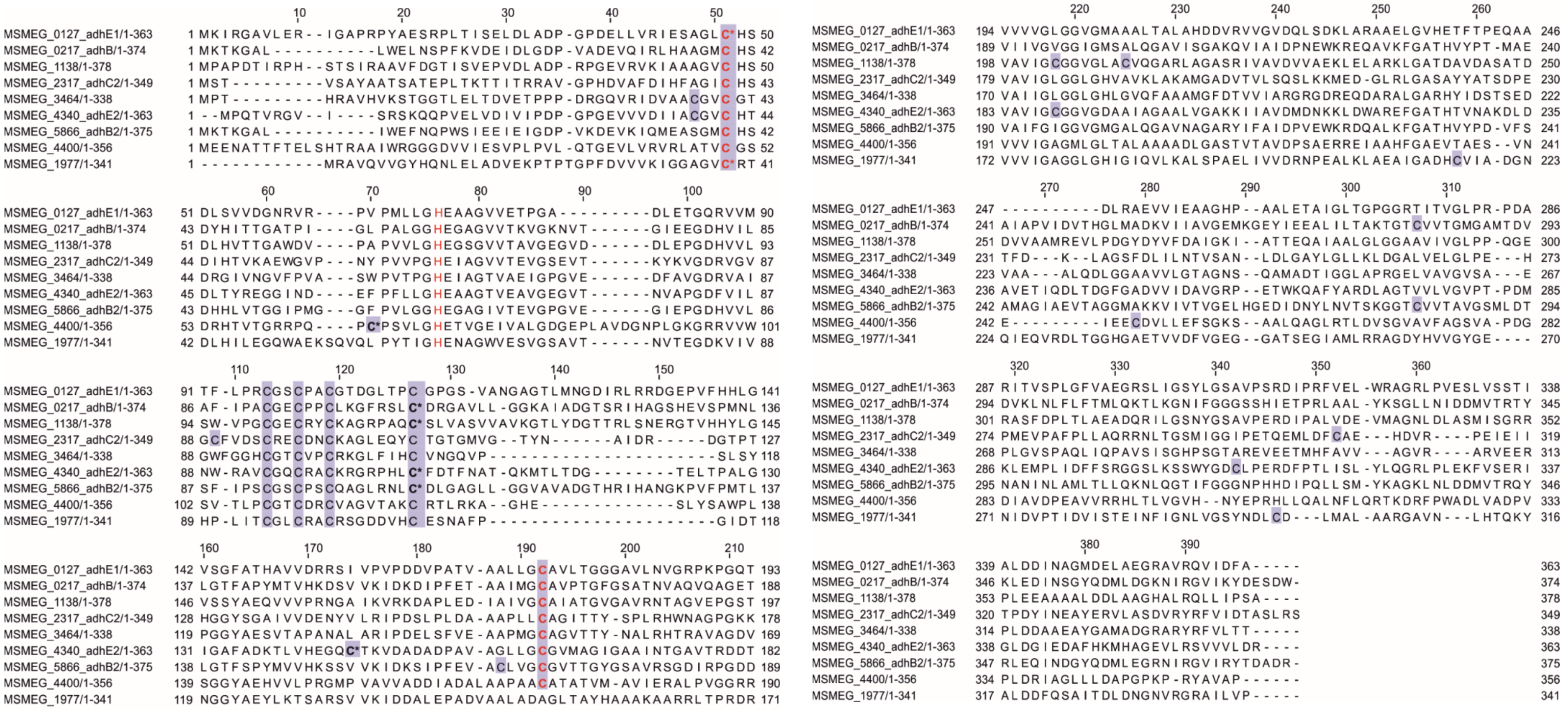
10 20 30 40 50
MSMEG_0127_adhE1/1-363 1 MKIRGAVLER--IGAPRPYAESRPLTISELDLADP-GPDELLVRIESAGLC*HS 50
MSMEG_0217_adhB/1-374 1 MKTKGAL----------LWELNSPFKVDEIDLGDP-VADEVQIRLHAAGMCHS 42
MSMEG_1138/1-378 1 MPAPDTIRPH--STSIRAAVFDGTISVEPVDLADP-RPGEVRVKIAAAGVCHS 50
MSMEG_2317_adhC2/1-349 1 MST---------VSAYAATSATEPLTKTTITRRAV-GPHDVAFDIHFAGICHS 43
MSMEG_3464/1-338 1 MPT---------HRAVHVKSTGGTLELTDVETPPP-DRGQVRIDVAACGVCGT 43
MSMEG_4340_adhE2/1-363 1 --MPQTVRGV--I----SRSKQQPVELVDIVIPDP-GPGEVVVDIIACGVCHT 44
MSMEG_5866_adhB2/1-375 1 MKTKGAL----------IWEFNQPWSIEEIEIGDP-VKDEVKIQMEASGMCHS 42
MSMEG_4400/1-356 1 MEENATTFTELSHTRAAIWRGGGDVVIESVPLPVL-QTGEVLVRVRLATVCGS 52
MSMEG_1977/1-341 1 ------------MRAVQVVGYHQNLELADVEKPTPTGPFDVVVKIGGAGVC*RT 41
60 70 80 90 100
MSMEG_0127_adhE1/1-363 51 DLSVVDGNRVR----PVPMLLGHEAAGVVETPGA---------DLETGQRVVM 90
MSMEG_0217_adhB/1-374 43 DYHITTGATPI----GLPALGGHEGAGVVTKVGKNVT------GIEEGDHVIL 85
MSMEG_1138/1-378 51 DLHVTTGAWDV----PAPVVLGHEGSGVVTAVGEGVD------DLEPGDHVVL 93
MSMEG_2317_adhC2/1-349 44 DIHTVKAEWGVP---NYPVVPGHEIAGVVTEVGSEVT------KYKVGDRVGV 87
MSMEG_3464/1-338 44 DRGIVNGVFPVA---SWPVTPGHEIAGTVAEIGPGVE------DFAVGDRVAI 87
MSMEG_4340_adhE2/1-363 45 DLTYREGGIND----EFPFLLGHEAAGTVEAVGEGVT------NVAPGDFVIL 87
MSMEG_5866_adhB2/1-375 43 DHHLVTGGIPMG---GFPVLGGHEGAGIVTEVGPGVE------GIEPGDHVVL 86
MSMEG_4400/1-356 53 DRHTVTGRRPQ----PC*PSVLGHETVGEIVALGDGEPLAVDGNPLGKGRRVVW 101
MSMEG_1977/1-341 42 DLHILEGQWAEKSQVQLPYTIGHENAGWVESVGSAVT------NVTEGDKVIV 88
110 120 130 140 150
MSMEG_0127_adhE1/1-363 91 TF-LPRCGSCPACGTDGLTPCGPGS-VANGAGTLMNGDIRLRRDGEPVFHHLG 141
MSMEG_0217_adhB/1-374 86 AF-IPACGECPPCLKGFRSLC*DRGAVLL-GGKAIADGTSRIHAGSHEVSPMNL 136
MSMEG_1138/1-378 94 SW-VPGCGECRYCKAGRPAQC*SLVASVVAVKGTLYDGTTRLSNERGTVHHYLG 145
MSMEG_2317_adhC2/1-349 88 GCFVDSCRECDNCKAGLEQYCTGTGMVG--TYN------AIDR-----DGTPT 127
MSMEG_3464/1-338 88 GWFGGHCGTCVPCRKGLFIHCVNGQVP------------------------SLSY 118
MSMEG_4340_adhE2/1-363 88 NW-RAVCGQCRACKRGRPHLC*FDTFNAT-QKMTLTDG--------TELTPALG 130
MSMEG_5866_adhB2/1-375 87 SF-IPSCGSCPSCQAGLRNLC*DLGAGLL-GGVAVADGTHRIHANGKPVFPMTL 137
MSMEG_4400/1-356 102 SV-TLPCGTCDRCVAGVTAKCRTLRKA--GHE-------------SLYSAWPL 138
MSMEG_1977/1-341 89 HP-LITCGLCRACRSGDDVHCESNAFP-----------------------GIDT 118
160 170 180 190 200 210
MSMEG_0127_adhE1/1-363 142 VSGFATHAVVDRRSIVPVPDDVPATV-AALLGCAVLTGGGAVLNVGRPKPGQT 193
MSMEG_0217_adhB/1-374 137 LGTFAPYMTVHKDSVVKIDKDIPFET-AAIMGCAVPTGFGSATNVAQVQAGET 188
MSMEG_1138/1-378 146 VSSYAEQVVVPRNGAIKVRKDAPLED-IAIVGCAIATGVGAVRNTAGVEPGST 197
MSMEG_2317_adhC2/1-349 128 HGGYSGAIVVDENYVLRIPDSLPLDA-AAPLLCAGITTY-SPLRHWNAGPGKK 178
MSMEG_3464/1-338 119 PGGYAESVTAPANALARIPDELSFVE-AAPMGCAGVTTY-NALRHTRAVAGDV 169
MSMEG_4340_adhE2/1-363 131 IGAFADKTLVHEGQC*TKVDADADPAV-AGLLGCGVMAGIGAAINTGAVTRDDT 182
MSMEG_5866_adhB2/1-375 138 LGTFSPYMVVHKSSVVKIDKSIPFEV-ACLVGCGVTTGYGSAVRSGDIRPGDD 189
MSMEG_4400/1-356 139 SGGYAEHVVLPRGMPVAVVADDIADALAAPAACATATVM-AVIERALPVGGRR 190
MSMEG_1977/1-341 119 NGGYAEYLKTSARSVVKIDDALEPADVAALADAGLTAYHAAAKAARRLTPRDR 171
220 230 240 250 260
MSMEG_0127_adhE1/1-363 194 VVVVGLGGVGMAAALTALAHDDVRVVGVDQLSDKLARAAELGVHETFTPEQAA 246
MSMEG_0217_adhB/1-374 189 VIIVGVGGIGMSALQGAVISGAKQVIAIDPNEWKREQAVKFGATHVVPT-MAE 240
MSMEG_1138/1-378 198 VAVIGCGGVGLACVQGARLAGASRIVAVDVVAEKLELARKLGATDAVDASATD 250
MSMEG_2317_adhC2/1-349 179 VAVIGLGGLGHVAVKLAKAMGADVTVLSQSLKKMED-GLRLGASAYYATSDPE 230
MSMEG_3464/1-338 170 VAIIGLGGLGHLGVQFAAAMGFDTVVIARGRGDREQDARALGARHYIDSTSED 222
MSMEG_4340_adhE2/1-363 183 VAVIGCGGVGDAAIAGAALVGAKKIIAVDMDNKKLDWAREFGATHTVNAKDLD 235
MSMEG_5866_adhB2/1-375 190 VAIFGIGGVGMGALQGAVNAGARYIFAIDPVEWKRDQALKFGATHVVPD-VFS 241
MSMEG_4400/1-356 191 VVVIGAGMLGLTALAAAADLGASTVTAVDPSAERREIAAHFGAEVTAES--VN 241
MSMEG_1977/1-341 172 VVVIGAGGLGHIGIQVLKALSPAELIVVDRNPEALKLAEAIGADHCVIA-DGN 223
270 280 290 300 310
MSMEG_0127_adhE1/1-363 247 ----------DLRAEVVIEAAGHP--AALETAIGLTGPGGRTITVGLPR-PDA 286
MSMEG_0217_adhB/1-374 241 AIAPVIDVTHGLMADKVIIAVGEMKGEYIEEALILTAKTGTCVVTGMGAMTDV 293
MSMEG_1138/1-378 251 DVVAAMREVLPDGYDYVFDAIGKI--ATTEQAIAALGLGGAAVIVGLPP-QGE 300
MSMEG_2317_adhC2/1-349 231 TFD---K--LAGSFDLILNTVSAN--LDLGAYLGLLKLDGALVELGLPE---H 273
MSMEG_3464/1-338 223 VAA---ALQDLGGAAVVLGTAGNS--QAMADTIGGLAPRGELVAVGVSA---E 267
MSMEG_4340_adhE2/1-363 236 AVETIQDLTDGFGADVVIDAVGRP--ETWKQAFYARDLAGTVVLVGVPT-PDM 285
MSMEG_5866_adhB2/1-375 242 AMAGIAEVTAGGMAKKVIVTVGELHGEDIDNYLNVTSKGGTCVVTAVGSMLDT 294
MSMEG_4400/1-356 242 E---------IEECDVLLEFSGKS--AALQAGLRTLDVSGVAVFAGSVA-PDG 282
MSMEG_1977/1-341 224 QIEQVRDLTGGHGAETVVDFVGEG--GATSEGIAMLRRAGDYHVVGYGE---- 270
320 330 340 350 360
MSMEG_0127_adhE1/1-363 287 RITVSPLGFVAEGRSLIGSYLGSAVPSRDIPRFVEL-WRAGRLPVESLVSSTI 338
MSMEG_0217_adhB/1-374 294 DVKLNLFLFTMLQKTLKGNIFGGGSSHIETPRLAAL-YKSGLLNIDDMVTRTY 345
MSMEG_1138/1-378 301 RASFDPLTLAEADQRILGSNYGSAVPERDIPALVDE-VMAGNLDLASMISGRR 352
MSMEG_2317_adhC2/1-349 274 PMEVPAFPLLAQRRNLTGSMIGGIPETQEMLDFCAE---HDVR----PEIEII 319
MSMEG_3464/1-338 268 PLGVSPAQLIQPAVSISGHPSGTAREVEETMHFAVV---AGVR----ARVEER 313
MSMEG_4340_adhE2/1-363 286 KLEMPLIDFFSRGGSLKSSWYGDCLPERDFPTLISL-YLQGRLPLEKFVSERI 337
MSMEG_5866_adhB2/1-375 295 NANINLAMLTLLQKNLQGTIFGGGNPHHDIPQLLSM-YKAGKLNLDDMVTRQY 346
MSMEG_4400/1-356 283 DIAVDPEAVVRRHLTLVGVH--NYEPRHLLQALNFLQRTKDRFPWADLVADPV 333
MSMEG_1977/1-341 271 NIDVPTIDVISTEINFIGNLVGSYNDLCD---LMAL-AARGAVN---LHTQKY 316
380 390
MSMEG_0127_adhE1/1-363 339 ALDDINAGMDELAEGRAVRQVIDFA----- 363
MSMEG_0217_adhB/1-374 346 KLEDINSGYQDMLDGKNIRGVIKYDESDW- 374
MSMEG_1138/1-378 353 PLEEAAALDDLAAGHALRQLLIPSA---- 378
MSMEG_2317_adhC2/1-349 320 TPDYINEAYERVLASDVRYRFVIDTASLRS 349
MSMEG_3464/1-338 314 PLDDAAEAYGAMADGRARYRFVLTT----- 338
MSMEG_4340_adhE2/1-363 338 GLDGIEDAFHKMHAGEVLRSVVVLDR---- 363
MSMEG_5866_adhB2/1-375 347 RLEQINDGYQDMLEGRNIRGVIRYTDADR- 375
MSMEG_4400/1-356 334 PLDRIAGLLLDAPGPKP-RYAVAP------ 356
MSMEG_1977/1-341 317 ALDDFQSAITDLDNGNVRGRAILVP----- 341

Fig. S1E

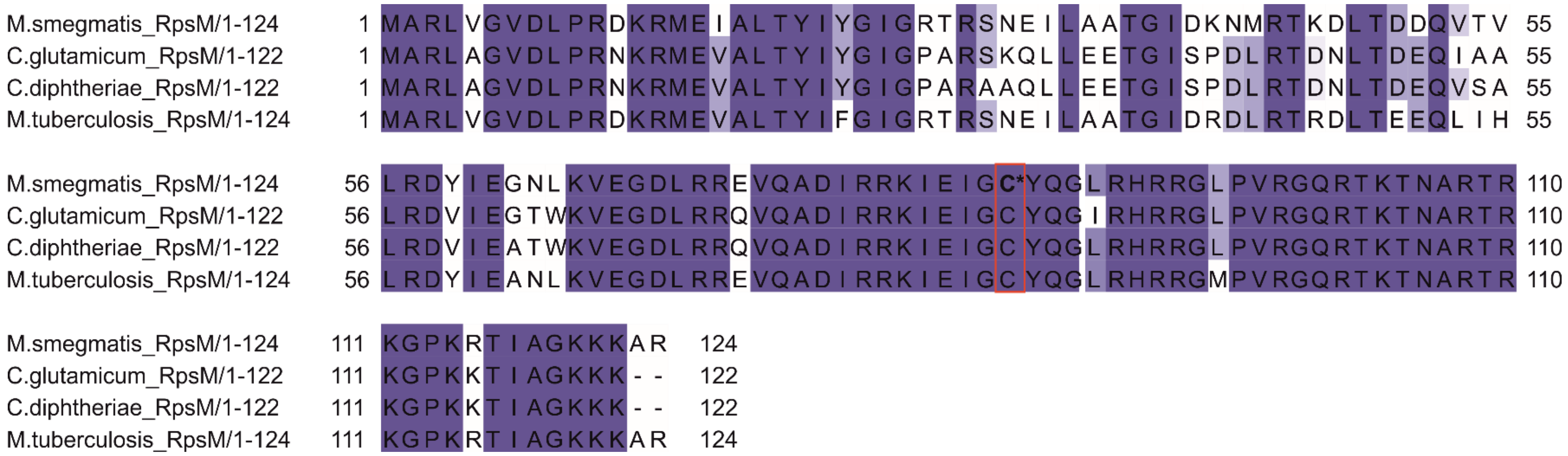
M.smegmatis_RpsM/1-124 1 MARLVGVDLPRDKRMEIALTYIYGIGRTRSNEILAATGIDKNMRTKDLTDDQVTV 55
C.glutamicum_RpsM/1-122 1 MARLAGVDLPRNKRMEVALTYIYGIGPARSKQLLEETGISPDLRTDNLTDEQIAA 55
C.diphtheriae_RpsM/1-122 1 MARLAGVDLPRNKRMEVALTYIYGIGPARAAQLLEETGISPDLRTDNLTDEQVSA 55
M.tuberculosis_RpsM/1-124 1 MARLVGVDLPRDKRMEVALTYIFGIGRTRSNEILAATGIDRDLRTRDLTEEQLIH 55
M.smegmatis_RpsM/1-124 56 LRDYIEGNLKVEGDLRREVQADIRRKIEIGC*YQGLRHRRGLPVRGQRTKTNARTR 110
C.glutamicum_RpsM/1-122 56 LRDVIEGTWKVEGDLRRQVQADIRRKIEIGCYQGIRHRRGLPVRGQRTKTNARTR 110
C.diphtheriae_RpsM/1-122 56 LRDVIEATWKVEGDLRRQVQADIRRKIEIGCYQGLRHRRGLPVRGQRTKTNARTR 110
M.tuberculosis_RpsM/1-124 56 LRDYIEANLKVEGDLRREVQADIRRKIEIGCYQGLRHRRGMPVRGQRTKTNARTR 110
M.smegmatis_RpsM/1-124 111 KGPKRTIAGKKKAR 124
C.glutamicum_RpsM/1-122 111 KGPKKTIAGKKK-- 122
C.diphtheriae_RpsM/1-122 111 KGPKKTIAGKKK-- 122
M.tuberculosis_RpsM/1-124 111 KGPKRTIAGKKKAR 124

Fig. S1F

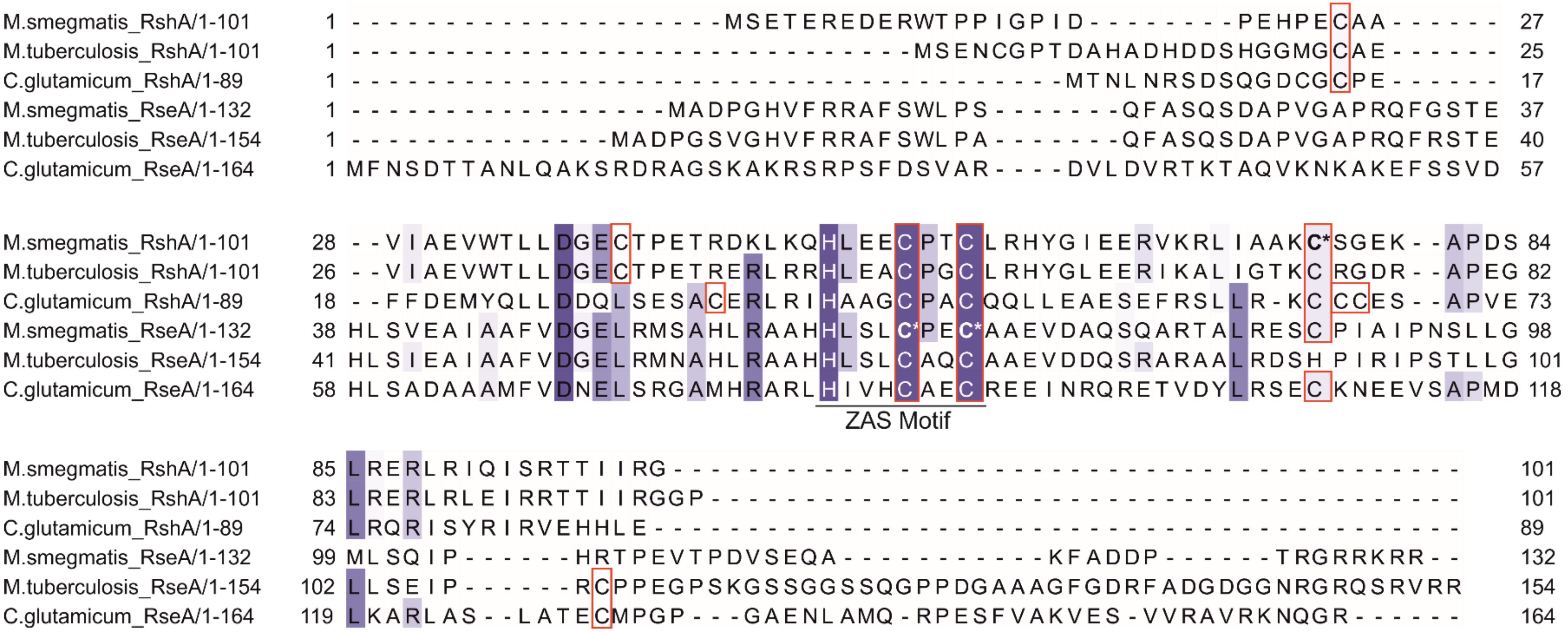
M.smegmatis_RshA/1-101 1 -------------------MSETEREDERWTPPIGPID--------PEHPECAA------ 27
M.tuberculosis_RshA/1-101 1 -----------------------------MSENCGPTDAHADHDDSHGGMGCAE------ 25
C.glutamicum_RshA/1-89 1 -------------------------------------MTNLNRSDSQGDCGCPE------ 17
M.smegmatis_RseA/1-132 1 ----------------MADPGHVFRRAFSWLPS-------QFASQSDAPVGAPRQFGSTE 37
M.tuberculosis_RseA/1-154 1 -------------MADPGSVGHVFRRAFSWLPA-------QFASQSDAPVGAPRQFRSTE 40
C.glutamicum_RseA/1-164 1 MFNSDTTANLQAKSRDRAGSKAKRSRPSFDSVAR---DVLDVRTKTAQVKNKAKEFSSVD 57
M.smegmatis_RshA/1-101 28 --VIAEVWTLLDGECTPETRDKLKQHLEECPTCLRHYGIEERVKRLIAAKC*SGEK--APDS 84
M.tuberculosis_RshA/1-101 26 --VIAEVWTLLDGECTPETRERLRRHLEACPGCLRHYGLEERIKALIGTKCRGDR--APEG 82
C.glutamicum_RshA/1-89 18 --FFDEMYQLLDDQLSESACERLRIHAAGCPACQQLLEAESEFRSLLR-KCCCES--APVE 73
M.smegmatis_RseA/1-132 38 HLSVEAIAAFVDGELRMSAHLRAAHHLSLC*PEC*AAEVDAQSQARTALRESCPIAIPNSLLG 98
M.tuberculosis_RseA/1-154 41 HLSIEAIAAFVDGELRMNAHLRAAHHLSLCAQCAAEVDDQSRARAALRDSHPIRIPSTLLG 101
C.glutamicum_RseA/1-164 58 HLSADAAAMFVDNELSRGAMHRARLHIVHCAECREEINRQRETVDYLRSECKNEEVSAPMD 118
ZAS Motif
M.smegmatis_RshA/1-101 85 LRERLRIQISRTTIIRG-- 101
M.tuberculosis_RshA/1-101 83 LRERLRLEIRRTTIIRGGP-- 101
C.glutamicum_RshA/1-89 74 LRQRISYRIRVEHHLE--- 89
M.smegmatis_RseA/1-132 99 MLSQIP------HRTPEVTPDVSEQA-----------KFADDP------TRGRRKRR-- 132
M.tuberculosis_RseA/1-154 102 LLSEIP------RCPPEGPSKGSSGGSSQGPPDGAAAGFGDRFADGDGGNRGRQSRVRR 154
C.glutamicum_RseA/1-164 119 LKARLAS--LATECMPGP---GAENLAMQ-RPESFVAKVES-VVRAVRKNQGR------ 164

Fig. S1G

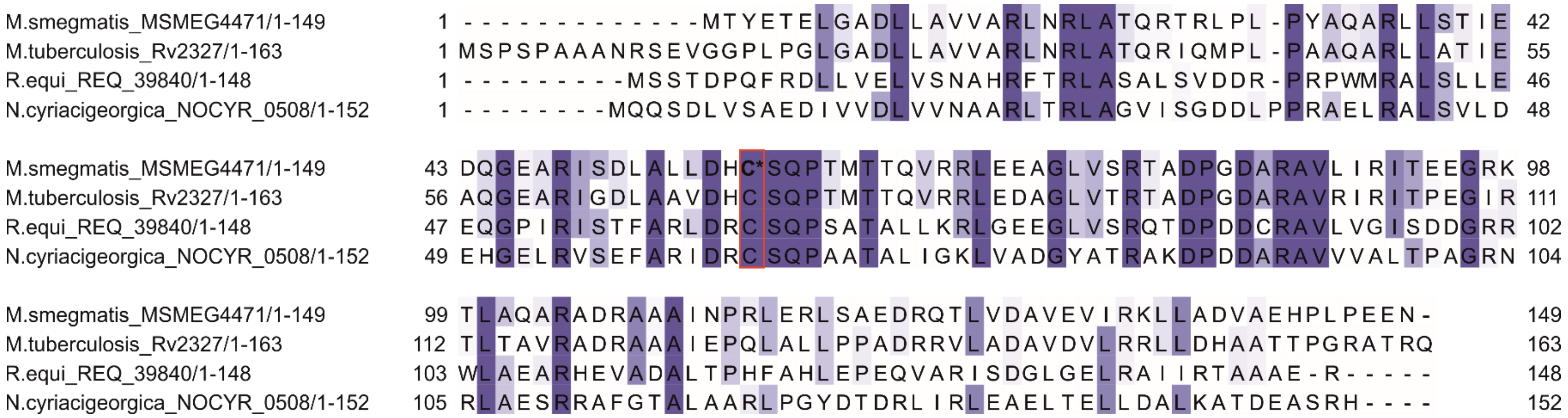

M.smegmatis_MSMEG4471/1-149 1 -------------MTYETELGADLLAVVARLNRLATQRTRLPL-PYAQARLLSTIE 42
M.tuberculosis_Rv2327/1-163 1 MSPSPAAANRSEVGGPLPGLGADLLAVVARLNRLATQRIQMPL-PAAQARLLATIE 55
R.equi_REQ_39840/1-148 1 ---------MSSTDPQFRDLLVELVSNAHRFTRLASALSVDDR-PRPWMRALSLLE 46
N.cyriacigeorgica_NOCYR_0508/1-152 1 --------MQQSDLVSAEDIVVDLVVNAARLTRLAGVISGDDLPPRAELRALSVLD 48
M.smegmatis_MSMEG4471/1-149 43 DQGEARISDLALLDHC*SQPTMTTQVRRLEEAGLVSRTADPGDARAVLIRITEEGRK 98
M.tuberculosis_Rv2327/1-163 56 AQGEARIGDLAAVDHCSQPTMTTQVRRLEDAGLVTRTADPGDARAVRIRITPEGIR 111
R.equi_REQ_39840/1-148 47 EQGPIRISTFARLDRCSQPSATALLKRLGEEGLVSRQTDPDDCRAVLVGISDDGRR 102
N.cyriacigeorgica_NOCYR_0508/1-152 49 EHGELRVSEFARIDRCSQPAATALIGKLVADGYATRAKDPDDARAVVVALTPAGRN 104
M.smegmatis_MSMEG4471/1-149 99 TLAQARADRAAAINPRLERLSAEDRQTLVDAVEVIRKLLADVAEHPLPEEN- 149
M.tuberculosis_Rv2327/1-163 112 TLTAVRADRAAAIEPQLALLPPADRRVLADAVDVLRRLLDHAATTPGRATRQ 163
R.equi_REQ_39840/1-148 103 WLAEARHEVADALTPHFAHLEPEQVARISDGLGELRAIIRTAAAE-R----- 148
N.cyriacigeorgica_NOCYR_0508/1-152 105 RLAESRRAFGTALAARLPGYDTDRLIRLEAELTELLDALKATDEASRH---- 152

Fig. S1H

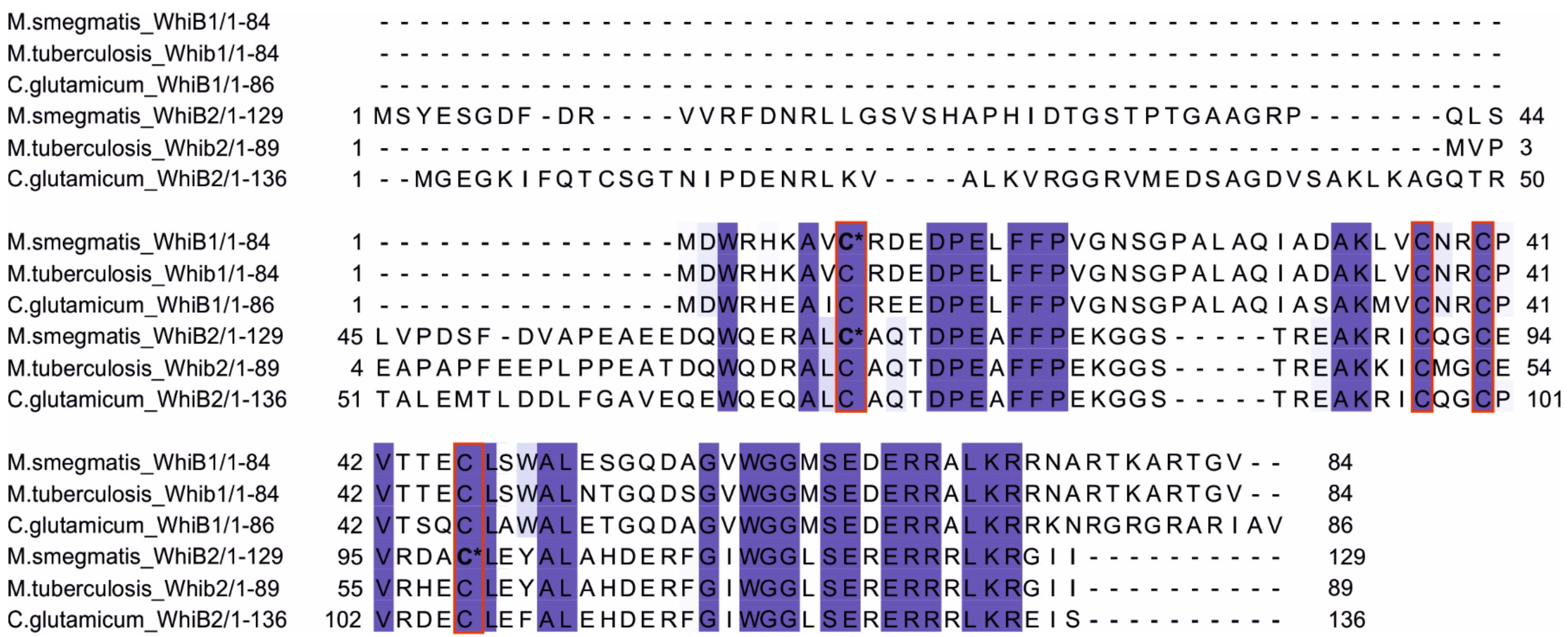
M.smegmatis_WhiB1/1-84 --
M.tuberculosis_Whib1/1-84 --
C.glutamicum_WhiB1/1-86 --
M.smegmatis_WhiB2/1-129 1 MSYESGDF-DR----VVRFDNRLLGSVSHAPHIDTGSTPTGAAGRP-------QLS 44
M.tuberculosis_Whib2/1-89 1 ---MVP 3
C.glutamicum_WhiB2/1-136 1 --MGEGKIFQTCSGTNIPDENRLKV----ALKVRGGRVMEDSAGDVSAKLKAGQTR 50
M.smegmatis_WhiB1/1-84 1 ---------------MDWRHKAVC*RDEDPELFFPVGNSGPALAQIADAKLVCNRCP 41
M.tuberculosis_Whib1/1-84 1 ---------------MDWRHKAVCRDEDPELFFPVGNSGPALAQIADAKLVCNRCP 41
C.glutamicum_WhiB1/1-86 1 ---------------MDWRHEAICREEDPELFFPVGNSGPALAQIASAKMVCNRCP 41
M.smegmatis_WhiB2/1-129 45 LVPDSF-DVAPEAEEDQWQERALC*AQTDPEAFFPEKGGS-----TREAKRICQGCE 94
M.tuberculosis_Whib2/1-89 4 EAPAPFEEPLPPEATDQWQDRALCAQTDPEAFFPEKGGS-----TREAKKICMGCE 54
C.glutamicum_WhiB2/1-136 51 TALEMTLDDLFGAVEQEWQEQALCAQTDPEAFFPEKGGS-----TREAKRICQGCP 101
M.smegmatis_WhiB1/1-84 42 VTTECLSWALESGQDAGVWGGMSEDERRALKRRNARTKARTGV-- 84
M.tuberculosis_Whib1/1-84 42 VTTECLSWALNTGQDSGVWGGMSEDERRALKRRNARTKARTGV-- 84
C.glutamicum_WhiB1/1-86 42 VTSQCLAWALETGQDAGVWGGMSEDERRALKRRKNRGRGRARIAV 86
M.smegmatis_WhiB2/1-129 95 VRDAC*LEYALAHDERFGIWGGLSERERRRLKRGII---------- 129
M.tuberculosis_Whib2/1-89 55 VRHECLEYALAHDERFGIWGGLSERERRRLKRGII---------- 89
C.glutamicum_WhiB2/1-136 102 VRDECLEFALEHDERFGIWGGLSERERRRLKREIS---------- 136

Figure S2

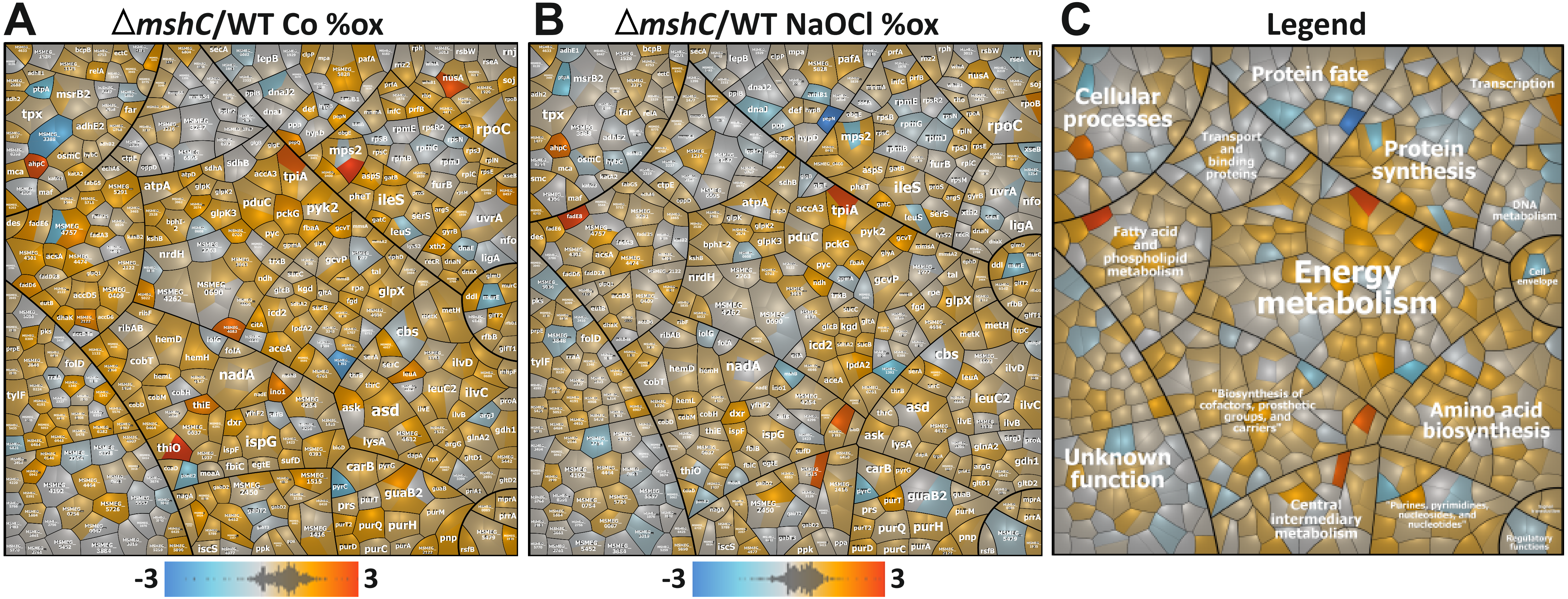

Fig. S2. Differential Voronoi redox treemaps for comparison of the thiol-oxidation level between the *M. smegmatis* wild type and the *mshC* mutant. The Voronoi redox treemaps visualize the log2 fold-change of the percentages of thiol-oxidations in the *mshC* mutant versus the wild type under control (A) and NaOCl stress conditions (B). The increased basal level oxidation in the mshC mutant control (A) is shown by the blue-yellow-red color gradient ranging from log2 fold-change oxidation of -3 to +3. The treemap in (C) is used as legend for the functional classification of the proteins displayed in (B). The treemaps were generated using the Paver software (Decodon) and proteins were classified according to the *M. smegmatis* TIGRfam annotation. The treemap results indicate that MSH is required to maintain the reduced state of the majority of protein thiols in *M. smegmatis*.
